# Protein sorting and proteostasis mechanisms in CFTR-related exocrine pancreas dysfunction: A systematic narrative review

**DOI:** 10.1080/19336950.2026.2731660

**Published:** 2026-09-12

**Authors:** Aime Patrick Niyomugabo, Leopold Ntakirutimana, Abdullateef Isiaka Alagbonsi

**Affiliations:** Department of Medical Physiology, School of Medicine and Pharmacy, College of Medicine and Health Sciences, University of Rwanda, Huye, Rwanda

**Keywords:** CFTR, channelopathies, exocrine pancreas, protein trafficking

## Abstract

The pancreas consists of exocrine and endocrine compartments. In the exocrine pancreas, cystic fibrosis transmembrane conductance regulator (CFTR) functions mainly in ductal epithelial cells as a chloride and bicarbonate channel. Its activity depends on proper protein folding, trafficking, and localization to the apical membrane. This systematic narrative review aims to synthesize the available evidence on the role of protein sorting machinery in CFTR channelopathies and its contribution to exocrine pancreatic dysfunction. A thorough search was conducted using PRISMA criteria on PubMed, Wiley Online Library, and Scopus for studies published in English between January 2000 and November 2025. Twenty studies that met the inclusion criteria were included in this review. Pathogenic CFTR variants impair protein folding, endoplasmic reticulum (ER) exit, and endosomal recycling, resulting in reduced apical membrane expression and stability. These defects disrupt the localization of associated transporters and secretory proteins, impair ductal bicarbonate secretion, alter zymogen handling, and promote acinar injury, although these claims are supported mainly by indirect experimental models and therefore require clinical confirmation. CFTR channelopathies in the exocrine pancreas encompass both ion transport defects and broader disruptions of protein sorting machinery. CFTR may contribute to the assembly, stabilization, or localization of selected apical transport complexes, and its loss can secondarily alter epithelial organization. Therapeutic approaches targeting both channel correction and intracellular trafficking may improve pancreatic function and mitigate disease progression.

## Introduction

The exocrine pancreas secretes approximately 2 liters per day of isotonic, bicarbonate-rich fluid, known as pancreatic juice, which contains multiple digestive enzymes mostly in their inactive form as zymogens. It is composed mainly of acinar cells (90%) that produce digestive enzymes, and ductal cells (10%) that secrete bicarbonate; a small number of mucus-secreting and endocrine cells are also present [1]. The pancreas and other organs such as the intestine, sweat glands, and airways express the cystic fibrosis transmembrane conductance regulator (CFTR), a chloride and bicarbonate channel on their ductal cell’s apical membrane that regulates salt, fluid, and ion transport across epithelial tissues and therefore plays important roles in fluid and electrolyte secretion and absorption [1,2]. CFTR is approximately 5 times more permeable to chloride (Cl^−^) than to bicarbonate (HCO_3_^−^), and provides Cl^−^ for the Cl^−^/HCO_3_^−^ exchanger, allowing HCO_3_^−^ to be secreted into the duct. CFTR also directly transports HCO_3_^−^, especially when high concentrations are needed. Activation by secretin (via cyclic adenosine monophosphate, cAMP) greatly increases this secretion, and water follows the ions, producing abundant alkaline pancreatic juice [3].

The CFTR is a member of the subfamily C, one of the seven subfamilies within the larger adenosine triphosphate (ATP)-binding cassette (ABC) transporter superfamily. It is composed of five total functional regions, including two transmembrane domains (TMD1 and TMD2), two cytoplasmic nucleotide-binding domains (NBD1 and NBD2), and an unstructured regulatory domain (RD) [4]. Each TMD has six transmembrane alpha helices (1–6 for TMD1 and 7–12 for TMD2), making a total of 12 structural helices spanning the lipid bilayer together [5,6]. Its glycoprotein is distinct because it is the only member of the large adenine nucleotide-binding cassette that acts as a passive ion channel, supporting the ATP-gated movement of ions (Cl^−^ and HCO_3_^−^) down their concentration gradient [3]. Its biogenesis involves co-translational insertion into the endoplasmic reticulum (ER), protein folding, ER quality control, Golgi maturation, and trafficking to the plasma membrane, where it functions as a cAMP-regulated Cl^−^ and HCO_3_^−^ channel [7]. CFTR surface expression is dynamically regulated by delivery, endocytosis, recycling, and degradation. These mechanisms include molecular chaperones, ubiquitin-proteasome components, and regulators of vesicular trafficking [8], all of which are essential for CFTR to arrive at the apical membrane correctly folded and able to function [9]. Mutations such as the deletion of phenylalanine at position 508 (F508del) disrupt folding and quality control, reducing functional CFTR at the cell surface and providing targets for therapeutic rescue [7]. Mutation-specific therapies remain unavailable for many rare CFTR mutations, prompting high-throughput screening and systems biology approaches to identify candidate compounds, clarify their mechanisms of action, and predict synergistic therapeutic combinations.

The CFTR lifecycle map provides a structured framework for understanding CFTR maturation, identifying therapeutic targets, and predicting mechanisms of candidate treatments [10]. The nucleus submap (encompassing 28 proteins, 16 RNAs and gene elements, and 1 small molecule/ion; accounting for 97% of interactors identified in the polarized cell) involves transcription of the *CFTR* gene into its mRNA. The ER submap (containing 45 proteins and 13 small molecules/ions; accounting for 69% of interactors identified in the polarized cell), encompasses the step-by-step translation of the mRNA into the CFTR peptide and its membrane integration, folding steps modulated by chaperones, core glycosylation, and the ER quality control process mediated by the calnexin cycle. Consequently, CFTR may proceed to the secretory pathway or be degraded by ER-associated degradation (ERAD). The secretory pathway submap (having 27 proteins and 8 small molecules/ions; accounting for 52% of interactors identified in the polarized cell) encompasses the COPII vesicle-mediated trafficking between the ER, Golgi, and the plasma membrane, and the full glycosylation at the Golgi apparatus. The activity submap (having 44 proteins and 20 small molecules/ions; accounting for 82% of interactors identified in the polarized cell) involves activation of CFTR by phosphorylation via cAMP signaling, channel opening and closing, and ion conductance. The final submap (having 23 proteins and accounting for 74% of interactors identified in the polarized cell) concerns the endocytosis of the mature CFTR protein from the plasma membrane, which can then either be recycled or degraded in lysosomes [10].

Cystic fibrosis (CF) is a lethal genetic disease caused by ~700 variations in the *CFTR* gene and remains one of the most common fatal hereditary disorders worldwide, afflicting about 85,000 people worldwide. Severe mutations, particularly classes I–III, are commonly associated with pancreatic insufficiency due to significant loss of CFTR function, whereas milder mutations, such as classes IV–VI, often retain partial CFTR activity and are more frequently linked to pancreatic sufficiency [11]. Channelopathies are a heterogeneous group of disorders resulting from the dysfunction of ion channels located in the membranes of all cells and many cellular organelles [9]. Defective CFTR (as in CF) leads to reduced HCO_3_^−^ and water secretion, resulting in thick pancreatic secretions, protein clogs, poor flushing of digestive enzymes, and duct obstruction. Consequently, these can lead to acinar cell damage, recurrent pancreatitis, and chronic pancreatic insufficiency [12].

Recently, we synthesized available evidence to show that the proper functioning of the gastric proton pump [13] and enterocytes [14] relies on precise protein sorting machinery that ensures correct delivery and recycling. We also demonstrated that defects in this sorting machinery result in gastric proton pumpopathy, causing impaired acid secretion due to issues like mislocalization or degradation of the pump [13]. Similarly, dysfunction of the enterocyte trafficking proteins underlies a group of severe disorders collectively termed sortopathies, including microvillus inclusion disease, chylomicron retention disease, and FHL5‑associated enteropathy [14]. However, beyond the genetic basis of CF associated with the mutation of the *CFTR* gene, little attention has been paid to CFTR channelopathies associated with abnormalities of the protein sorting machinery in the ductal cells. Most existing studies have primarily focused on its ion transport function in the pathogenesis of CF, even though emerging evidence indicates that CFTR dysfunction is also closely linked to abnormalities in intracellular protein folding, trafficking, and apical membrane targeting, processes governed by complex protein sorting machinery. Disruptions in these pathways contribute not only to defective channel activity but also to mislocalization of associated transporters and secretory proteins, particularly in the exocrine pancreas.

Although individual studies have explored aspects of CFTR trafficking and its impact on pancreatic function, the evidence remains fragmented and lacks comprehensive synthesis. Therefore, this review focuses on how protein sorting machinery abnormalities contribute to CFTR channelopathies in the exocrine pancreas by examining the roles of molecular chaperones, ubiquitin ligases, deubiquitinating enzymes, and vesicular trafficking systems in CFTR maturation and stability, highlighting the mechanistic links between intracellular sorting defects and pancreatic pathology. Understanding these relationships is essential for developing therapeutic strategies that rescue CFTR processing and restore normal exocrine pancreatic function. This objective sought to answer the research question: What is the role of intracellular protein sorting mechanisms in regulating CFTR trafficking and function, and how do defects in these processes contribute to exocrine pancreatic dysfunction in CFTR-related disease?

## Methodology

### Study design

The review employed the PRISMA (Preferred Reporting Items for Systematic Reviews and Meta-Analyses) 2020 guidelines for reporting [15], as adopted in previous studies that combined narrative synthesis with systematic reviews [16–26]. The PRISMA 2020 checklist can be found in the Supplementary File S1.

### Search strategy

A comprehensive literature search was performed using the Wiley Online Library, PubMed, and Scopus, covering studies published from 1 January 2000 to 30 November 2025. The search targeted research investigating CFTR channelopathies caused by abnormalities in the protein sorting and trafficking machinery within the exocrine pancreas. The search strategy combined controlled vocabulary and free-text keywords. Core terms related to CFTR dysfunction included: “CFTR,” “CFTR mutations,” “CFTR channelopathies,” “cystic fibrosis transmembrane conductance regulator,” “CFTR misfolding,” and “CFTR trafficking defects.” To capture the role of intracellular sorting systems, we used protein-processing terms such as “protein sorting,” “protein trafficking,” “endoplasmic reticulum,” “Golgi apparatus,” “ER-associated degradation (ERAD),” “chaperone proteins,” “COPII vesicles,” “quality control machinery,” and “ubiquitin-proteasome pathway.” Because the focus is on the exocrine pancreas, organ-specific keywords were included: “exocrine pancreas,” “pancreatic acinar cells,” “pancreatic ductal cells,” “pancreatic secretion,” and “bicarbonate secretion.” To ensure broad coverage of experimental approaches, additional search terms were incorporated for study models such as “animal models,” “mouse models,” “rat models,” “organoids,” “cell lines,” and “*in vitro* studies.” To ascertain whether additional eligible studies could be identified, manual searches and reference-list screening were used as supplementary methods. The search strings used for each database are presented in the Supplementary File S2.

### Inclusion and exclusion criteria

Studies were selected based on predefined inclusion and exclusion criteria to ensure that only relevant and high-quality literature addressing CFTR channelopathies and their relationship with protein sorting machinery in the exocrine pancreas was considered. Included studies were those published between 1 January 2000 and 30 November 2025, written in English, and focused on CFTR mutations, misfolding, or trafficking defects within the context of protein sorting pathways such as the ER, Golgi apparatus, ERAD, and ubiquitin-proteasome systems. Eligible studies examined exocrine pancreatic tissue, particularly acinar and ductal cells, and included experimental models such as animal studies, *in vitro* systems, organoids, and mechanistic human studies. Studies were excluded if they primarily investigated CFTR function in non-pancreatic organs without direct relevance to pancreatic protein trafficking, focused solely on clinical manifestations of CF without mechanistic analysis, or centered on the endocrine pancreas only. Commentaries, conference abstracts, opinion pieces, non-peer-reviewed materials, and review articles were also excluded, as were studies lacking full text or adequate methodological detail (Table 1).

**Table 1.** Inclusion and exclusion criteria for articles.

Category	Inclusion Criteria	Exclusion Criteria
Topic Relevance	Studies on CFTR mutations, misfolding, trafficking defects, or channelopathies involving protein sorting or quality control mechanisms in the exocrine pancreas	Studies focusing on CFTR in other organs (e.g. lungs, intestine, sweat glands) without pancreatic relevance; endocrine pancreas studies only
Mechanistic Focus	Research examining ER/Golgi processing, ERAD, chaperones, COPII trafficking, or proteasomal degradation related to CFTR	Studies addressing CFTR only from electrophysiological or clinical perspectives, with no protein sorting component
Study Types	Experimental studies, in vitro work, animal models, organoids, human tissue studies	Editorials, commentaries, letters, conference abstracts, non-peer-reviewed sources, and review articles.
Publication Date	1 January 2000– 30 November 2025	Published outside the date range
Language	English	Non-English publications
Availability	Full-text accessible	Studies without an available full text or insufficient methodological detail

### Data extraction and analysis

From each included study, the following data were extracted from the full text: (i) first author and year of publication, (ii) study type (animal model, cellular model, or human study), (iii) detailed characteristics of the experimental model (e.g. Wistar rats, transgenic mice, pancreatic ductal cell lines, organoids, or human pancreatic tissue), (iv) type of intervention or experimental manipulation (such as ligand administration, genetic modification, receptor blockade, or knockout models), (v) target tissues or cellular compartments examined (e.g. exocrine pancreas, pancreatic ductal cells, acinar cells, ER–Golgi axis), (vi) key outcome measures (including CFTR expression, trafficking efficiency, protein folding status, ER stress markers, secretion function, or related signaling pathways), and (vii) proposed mechanistic pathways involved (such as ERAD engagement, chaperone interactions, COPII transport, ubiquitin–proteasome degradation, or misfolding-related signaling cascades. The detailed data were extracted using a piloted and validated form [27–33] and are available as Supplementary File S3. All extracted data were cross-checked by all reviewers. Any discrepancies in interpretation or classification were resolved through joint discussion until consensus was reached. Risk of bias in the included studies was assessed using structured appraisal tools appropriate for each study type: Systematic Review Center for Laboratory Animal Experimentation (SYRCLE)’s Risk of Bias tool for animal studies [34], human studies were assessed using the Cochrane RoB 1 tool [35], while in vitro studies were assessed using the modified Checklist for Reporting In Vitro Studies (CRIS) [36], emphasizing experimental design, reproducibility, control conditions, and statistical rigor. The risk of bias report of the included studies is also presented in Supplementary File S4.

### Mechanistic categorization and thematic analysis

Findings were synthesized by categorizing CFTR dysfunction according to key mechanistic levels in exocrine pancreatic cells. Central themes included ER protein folding and quality control, ER–Golgi trafficking, apical membrane targeting, and degradation pathways such as ERAD and proteasomal clearance. Studies were also grouped by physiological or pathological context, including normal pancreatic function, CFTR-mutant models, and stress-induced misfolding conditions. Mechanistic pathways, including chaperone-mediated folding, vesicular transport (COPII/COPI), and kinase regulation, were mapped to understand defects in CFTR processing and secretion. Contradictory findings, such as partial rescue of misfolded CFTR versus persistent trafficking failure, were noted. Gaps were identified in mutation-specific trafficking, interactions with proteostasis networks, and links to exocrine secretion. Overall, results were integrated into functional domains encompassing folding/quality control, trafficking, membrane stability, degradation, and exocrine pancreatic function.

## Results

**Figure 1.**
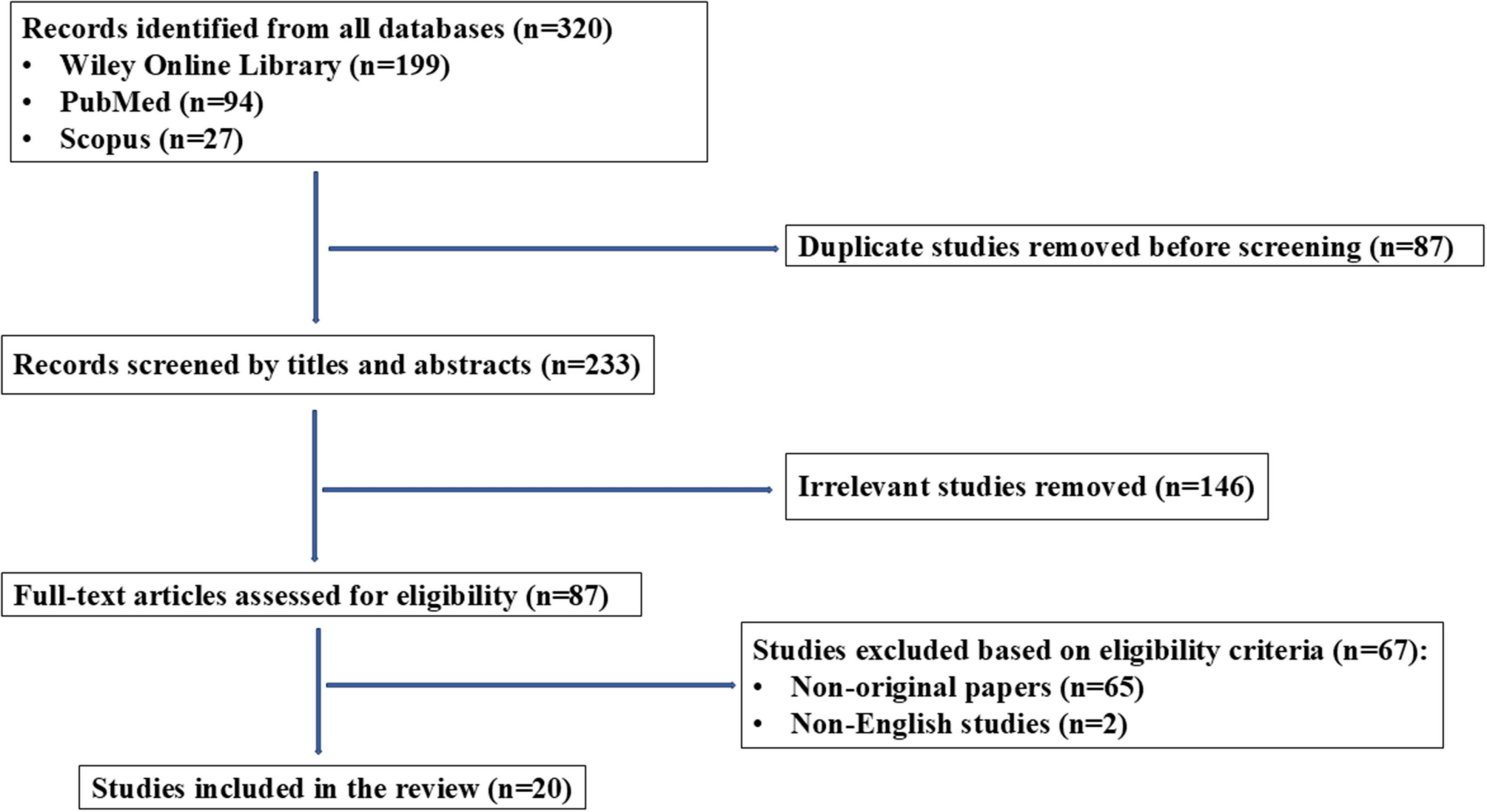
PRISMA flow chart for the article.

A total of 320 records were identified from all databases, including 199 from Wiley Online Library, 94 from PubMed, and 27 from Scopus. Supplementary manual searches and reference-list screening did not yield any unique studies. Out of these 320 records, 87 duplicates were removed before screening using Mendeley Reference Manager, yielding 233 records that were screened by title and abstract. During this initial screening, 146 studies that were not relevant to the study objective were removed, yielding 87 full-text articles. During this second screening, 67 studies were excluded, including 65 non-original papers and 2 non-English studies. Finally, 20 original studies that met the inclusion criteria were retained for the synthesis of evidence in this systematic review (Figure 1). Various domains were identified, including ER quality control and ERAD, Golgi maturation and vesicular trafficking, ubiquitin-mediated degradation pathways, endocytic recycling and membrane stability, and regulatory interactors that modulate CFTR function (Table 2).

**Table 2.** Summary of information from the included studies.

Categories	Authors	Experimental model	Key findings	Level of relevance
ER quality control and ERAD	Ballar et al.[37]	Human cell culture expressing mutant CFTR (ΔF508)	gp78 promotes ubiquitination and ERAD degradation of mutant CFTR (ΔF508). Silencing gp78 or inhibiting it with SVIP leads to the accumulation of CFTRΔF508. Hrd1 regulates gp78 stability and indirectly modulates CFTR degradation	Indirect non-pancreatic CFTR evidence
	Hocevar et al.[38]	Mouse pancreatic acinar cell	PFOA activates UPR in pancreatic acinar cells and points to ER stress as a central stress response. In pancreatic epithelia, where CFTR is critical, UPR activation and the resulting Ca^2+^ dysregulation can undermine CFTR folding and trafficking. This mechanistic overlap suggests that environmental UPR triggers (like PFOA) could indirectly influence CFTR function and contribute to pancreatic secretory dysfunction, a key feature of CFTR‑related pancreatic disease	General pancreatic proteostasis evidence
	Ludovico and Baroni [39]	CFPAC‑1 pancreatic ductal epithelial cell line expressing F508del CFTR mutation	CFTR function was improved by modulating the intracellular biosynthetic trafficking pathway of the mutant F508del-CFTR protein. The corrector components of the triple CFTR modulator therapy (VX-661 and VX-445) promoted proper folding of the misfolded CFTR in the ER, enabling it to escape ER quality control and avoid premature degradation. This facilitated forward trafficking from the ER through the Golgi apparatus to the apical plasma membrane, increasing the number of functional CFTR channels at the cell surface. As a result, treatment with the triple combination (VX-770/VX-661/VX-445) enhanced CFTR-mediated ion and fluid transport, increased apical surface fluid pH, and reduced microviscosity in CFPAC-1 pancreatic epithelial cells.	Direct pancreatic CFTR evidence
	Riepe et al. [40]	Human cell lines expressing F508del CFTR	Small-molecule correctors (tezacaftor + elexacaftor) stabilize sequential CFTR folding states, reducing ERAD and enhancing trafficking to the plasma membrane	Indirect non-pancreatic CFTR evidence
	Navis and Bagnat [41]	Zebrafish (*Danio rerio*) larval model with a cftr mutant line (cftr^pd1049/pd1049) — loss‑of‑function genetic model.	Loss of CFTR function leads to severe pancreatic destruction in zebrafish larvae, demonstrating its essential role in the development and maintenance of the exocrine pancreas. Defective intracellular trafficking—particularly misfolding and retention of CFTR in the endoplasmic reticulum prevents the protein from reaching the apical membrane of ductal epithelial cells. As a result, chloride and bicarbonate secretion are impaired, leading to reduced fluid secretion, thickened pancreatic secretions, ductal obstruction, and subsequent acinar cell injury and tissue degeneration.	Direct pancreatic CFTR evidence
	Gnann et al. [42]	Saccharomyces cerevisiae (yeast) expressing CFTR	The ERAD of misfolded CFTR is a coordinated multi-step process beginning with the recognition of the protein by the lectin-like Htm1p/EDEM, which identifies misfolded glycans and prevents their premature exit from the ER. Once identified, the E3 ubiquitin ligases Hrd1p (Der3p) and Doa10p work together to attach ubiquitin chains to the CFTR substrate, marking it for destruction. Finally, the Cdc48p (p97) complex acts as a molecular motor, providing the necessary mechanical force to extract the polyubiquitinated CFTR from the ER membrane and deliver it to the cytosol for degradation by the 26S proteasome.	Indirect non-pancreatic CFTR evidence
Golgi Maturation and Vesicular Trafficking	Kostenko et al.[43]	Mouse pancreatic acinar cells (primary acini and genetically manipulated mouse models); ex vivo and in vivo analyses	c-Src kinase regulates cargo transit through the Golgi apparatus in pancreatic acinar cells; dysregulation of this pathway alters secretory trafficking and may contribute to pancreatic pathology	General pancreatic proteostasis evidence
	Lee and Goldberg [44]	Purified protein complexes from *Bos taurus* (bovine) and subcomplexes expressed in insect cells (baculovirus system) and *E. coli* for crystallography. This is a biochemical/structural model, not a cell‑based or animal pathology model	Reveals conserved architectural principles among COPI, COPII, and clathrin vesicle coats, demonstrating a shared evolutionary origin and providing a structural framework for understanding vesicle formation and cargo sorting	Background cell biology evidence
	Rizzo et al.[45]	*In vitro* human cell systems — primarily HeLa cells; also primary human fibroblasts (PHFs)	GOLPH3 binds specific glycosphingolipid (GSL) biosynthetic enzymes through a cytosolic LxxR motif, sorting them into COPI vesicles for retrograde intra-Golgi transport. Acting within the Golgi cisternal maturation process, GOLPH3 controls enzyme localization and retention in the trans-Golgi and prevents their lysosomal degradation. Elevated GOLPH3 increases complex GSL synthesis, alters plasma membrane composition, and promotes mitogenic signaling via pathways such as Akt/mTOR, thereby supporting cell growth.	Background cell biology evidence
	Hollande et al.[46]	Human pancreatic duct CFPAC-1 cells (CFTR ΔF508 mutation) and CFPAC-1 cells transfected with wild-type CFTR to generate a “reverted” phenotype expressing normal CFTR.	CFPAC-1 cells expressing ΔF508 CFTR exhibit fragmented and dispersed Golgi stacks accompanied by impaired intracellular protein trafficking, including defective routing of carbonic anhydrase IV. Reintroduction of wild-type CFTR restores normal Golgi architecture, microtubule organization, and secretory pathway function, highlighting a critical role for CFTR in maintaining biosynthetic and secretory pathway integrity in pancreatic duct cells	Direct pancreatic CFTR evidence
	Dahan et al.[47]	Primary pancreatic acinar cells isolated from rodents (ex vivo cell model) were stimulated with secretagogues such as cholecystokinin (CCK) to mimic physiologic secretory stimulus.	CCK‑induced secretory stimulation causes selective fragmentation/vesiculation of the trans‑Golgi network (TGN), correlating with increased cytoplasmic Ca^2+^, and dynamin is recruited to the Golgi, where it promotes TGN fragmentation, suggesting dynamic regulation of Golgi architecture during regulated secretion	General pancreatic proteostasis evidence
Ubiquitin-Mediated Degradation Pathways	Younger et al.[48]	Cultured mammalian cells (HEK293 transfected with CFTR and mutants)	Identified sequential ER quality‑control checkpoints involving ER membrane‑associated RMA1/Ubc6e/Derlin‑1 complex cooperating with cytosolic Hsc70/CHIP to recognize and target misfolded CFTR, especially ΔF508, for proteasomal degradation. RMA1 acts cotranslationally while CHIP acts post‑translationally. Derlin‑1 helps retain misfolded CFTR in the ER. This defines how cells triage misfolded CFTR for degradation	Indirect non-pancreatic CFTR evidence
	Okiyoneda et al.[49]	Human cell lines (e.g. HeLa and IB3 cells) expressing rescued ΔF508-CFTR; post‑Golgi compartments studied in vitro and in cultured cells	Shows that unfolded CFTR that escapes the ER is recognized and removed at the plasma membrane by peripheral quality-control mechanisms; highlights an additional layer beyond ERAD that limits functional CFTR at the cell surface	Indirect non-pancreatic CFTR evidence
	Jarosch et al.[50]	*Saccharomyces cerevisiae* (yeast) cellular system, studying ER‑associated protein degradation pathways	Demonstrates that ER protein dislocation requires polyubiquitination and the AAA-ATPase Cdc48, providing mechanistic insight into the retrotranslocation step of ERAD	Background cell biology evidence
	Wu et al.[51]	Human pancreatic cancer cell lines (e.g. Panc-1, PaTu-8988), 293T cells, and xenograft mouse models	Fbxo45 interacts with and ubiquitinates USP49 via its SPRY domain, promoting degradation of USP49, enhancing pancreatic cancer cell proliferation, migration, invasion, and tumor growth in vivo; Fbxo45 expression is negatively correlated with USP49 and associated with poorer survival	General pancreatic proteostasis evidence
Endocytic Recycling and Membrane Stability	Heda et al.[52]	Polarized epithelial cell culture (MDCK cells expressing wild-type or ΔF508-CFTR)	ΔF508-CFTR that reaches the plasma membrane in polarized epithelial cells exhibits a significantly shortened biochemical half-life compared with wild-type CFTR, indicating enhanced post-Golgi instability and accelerated removal from the cell surface. This defect contributes to reduced functional CFTR at the apical membrane in cystic fibrosis	Indirect non-pancreatic CFTR evidence
	Sharma et al.[53]	Mammalian cell culture (primarily human epithelial cell lines expressing CFTR, including wild-type and ΔF508-CFTR)	Misfolded CFTR that escapes the ER quality-control system is rapidly removed from the plasma membrane and diverted from the recycling pathway to lysosomal degradation. In contrast, properly folded CFTR efficiently recycles back to the cell surface. This demonstrates the existence of a post-ER peripheral quality-control mechanism that recognizes misfolded CFTR and targets it for degradation, contributing significantly to CFTR loss in cystic fibrosis.	Indirect non-pancreatic CFTR evidence
	Bomberger et al.[54]	Human airway epithelial cells (CFBE cells)	USP10 localizes to early endosomes and deubiquitinates CFTR, promoting its recycling back to the plasma membrane. Knockdown of USP10 increases CFTR ubiquitination and lysosomal degradation and decreases CFTR at the apical membrane and CFTR-mediated chloride secretion, while overexpression of wild-type USP10 enhances CFTR abundance and reduces its ubiquitination.	Indirect non-pancreatic CFTR evidence
Regulatory Interactors Modulating CFTR Function	Angyal et al.[55]	Adult stem cell‑derived organoids from porcine pancreas, differentiated into ductal epithelial monolayers	Shows that pro-inflammatory cytokines enhance CFTR-dependent anion (Cl^−^ and HCO_3_^−^) secretion, indicating that inflammation can modulate CFTR function in pancreatic ducts and potentially influence pancreatic pathology	Direct pancreatic CFTR evidence
	Kubisch et al.[56]	Isolated rat pancreatic acinar cells	All secretagogues increased BiP and XBP1 splicing, activating adaptive ER stress, whereas only high-dose CCK8 triggered PERK	General pancreatic proteostasis evidence

Abbreviations: AAA-ATPase, ATPases associated with diverse cellular activities; Akt/mTOR, protein kinase B/mammalian target of rapamycin signaling pathway; BiP, binding immunoglobulin protein; Ca^2+^, calcium ion; CCK/CCK8, cholecystokinin; Cdc48p (p97), ATPase involved in protein extraction from the endoplasmic reticulum; CFBE, cystic fibrosis bronchial epithelial cells; CFPAC-1, human pancreatic ductal adenocarcinoma cell line; CFTR, cystic fibrosis transmembrane conductance regulator; CHIP, C-terminus of Hsc70-interacting protein; Cl^−^, chloride ion; COPI, coat protein complex I; COPII, coat protein complex II; ΔF508/F508del, deletion of phenylalanine at position 508 in CFTR; Derlin1, ER membrane protein involved in retro-translocation; Doa10p, ER membrane ubiquitin ligase;*E. coli,Escherichia coli*; EDEM, ER degradation-enhancing α-mannosidase-like protein; ER, endoplasmic reticulum; ERAD, endoplasmic reticulum-associated degradation; Fbxo45, F-box protein 45; GOLPH3, Golgi phosphoprotein 3; GSL, glycosphingolipid; HCO_3_^−^, bicarbonate ion; HEK293/293T, human embryonic kidney cells; HeLa, human cervical cancer cells; Hrd1, E3 ubiquitin ligase; Hsc70, heat shock cognate 70; Htm1p, homologous to mannosidase 1 protein; IB3, human bronchial epithelial cystic fibrosis cell line; LxxR motif, leucine–any–any–arginine motif; MDCK, Madin–Darby canine kidney cells; Panc-1, human pancreatic cancer cell line; PaTu-8988, human pancreatic cancer cell line; PERK, protein kinase RNA-like endoplasmic reticulum kinase; PFOA, perfluorooctanoic acid; PHFs, primary human fibroblasts; RMA1, ring finger membrane-anchor protein 1; SPRY domain, spla and ryanodine receptor domain; SVIP, small VCP-interacting protein; TGN, trans-Golgi network; Ubc6e, ubiquitin-conjugating enzyme E2; UPR, unfolded protein response; USP10, ubiquitin-specific protease 10; USP49, ubiquitin-specific protease 49; VX-445, elexacaftor; VX-661, tezacaftor; VX-770, ivacaftor; XBP1, X-box binding protein 1.

### Data synthesis

#### ER quality control and ERAD

Evidence from previous studies indicated that ER quality control determines whether newly synthesized proteins are competent to proceed through the secretory pathway, whereas ERAD functions as a disposal mechanism that eliminates proteins that fail folding checkpoints, thereby preserving ER homeostasis and overall cellular health [39]. The ER works as a multifunctional organelle where it coordinates protein synthesis, folding, quality control, trafficking, calcium homeostasis, and detoxification, and the disruption of these functions by perfluorooctanoic acid (PFOA) has been shown to induce ER stress–mediated activation of the PERK, IRE1α, and activating transcription factor 6 (ATF6) unfolded protein response (UPR) pathways [38]. The ΔF508 mutation in the CFTR gene causes protein misfolding, leading to enhanced ERAD and reduced trafficking to the cell surface [39]. The CFTR correctors tezacaftor (VX-661) and elexacaftor (VX-445) rescue F508del-CFTR by stabilizing key folding intermediates and reducing ERAD, thereby clarifying the molecular mechanism underlying effective CFTR modulator therapy [40]. Loss or misfolding of CFTR disrupts ER quality control and triggers ERAD, preventing functional CFTR from reaching the plasma membrane and leading to pancreatic pathology [41]. Silencing of the ERAD-associated E3 ligase gp78 increases CFTRΔF508 accumulation by reducing its ubiquitination and interaction with p97/VCP, while Hrd1 indirectly promotes CFTRΔF508 stability by targeting gp78 for degradation, highlighting a regulatory interplay between gp78 and Hrd1 in CFTRΔF508 ERAD [37]. CFTR ΔF508 undergoes ERAD-mediated degradation via Htm1p/EDEM, Der3p/Hrd1p, Doa10p, and the Cdc48p-Ufd1p-Npl4p complex, illustrating a conserved ER quality control pathway driving CFTR channelopathy [42]. In the pancreas, high secretory demand makes ER quality control and ERAD essential for directing properly folded proteins forward, eliminating misfolded species, limiting ER stress, and preserving β-cell and acinar cell function.

### Golgi maturation and vesicular trafficking

The available evidence highlights the role of dysregulation of Golgi maturation and vesicular trafficking processes in CFTR pancreatic channelopathies. The exocrine pancreas is a highly specialized secretory tissue that relies on effective Golgi maturation and vesicular trafficking to maintain the vast synthesis and controlled secretion of digestive enzymes. Activation of c-Src in pancreatic acinar cells enhances Golgi expansion and promotes zymogen granule formation and protein trafficking, whereas its inhibition disrupts Golgi structure, reduces zymogen granule content, and causes retention of digestive enzymes such as amylase within microsomal compartments and the endoplasmic reticulum [43]. In exocrine pancreatic acinar cells, elevated GOLPH3 disrupts intra-Golgi localization of glycosphingolipid-synthesizing enzymes, reshaping membrane composition and receptor signaling to drive mitogenic activity, dysregulated secretion, and enhanced proliferation [45]. Also, dysregulated secretion triggers calcium-dependent fragmentation of the trans-Golgi network with intact pre-TGN and constitutive pathways and is enhanced by dynamin recruitment, which may impair CFTR maturation and apical trafficking, thereby reducing its surface expression and chloride channel function [47]. Disruption of COPI-mediated anterograde and retrograde trafficking impairs CFTR recycling, linking defects in coatomer function to CFTR channelopathy and mislocalization [44]. Ultrastructural and immunocytochemical analyses showed that expression of ΔF508 CFTR in polarized CFPAC-1 cells causes Golgi fragmentation and dispersal associated with abnormal microtubule organization and multiple MTOCs, leading to disruption of the biosynthetic/secretory pathway and defective intracellular transport of CA IV, all of which are restored in reverted cells [46].

### Ubiquitin-mediated degradation pathways

**Figure 2.**
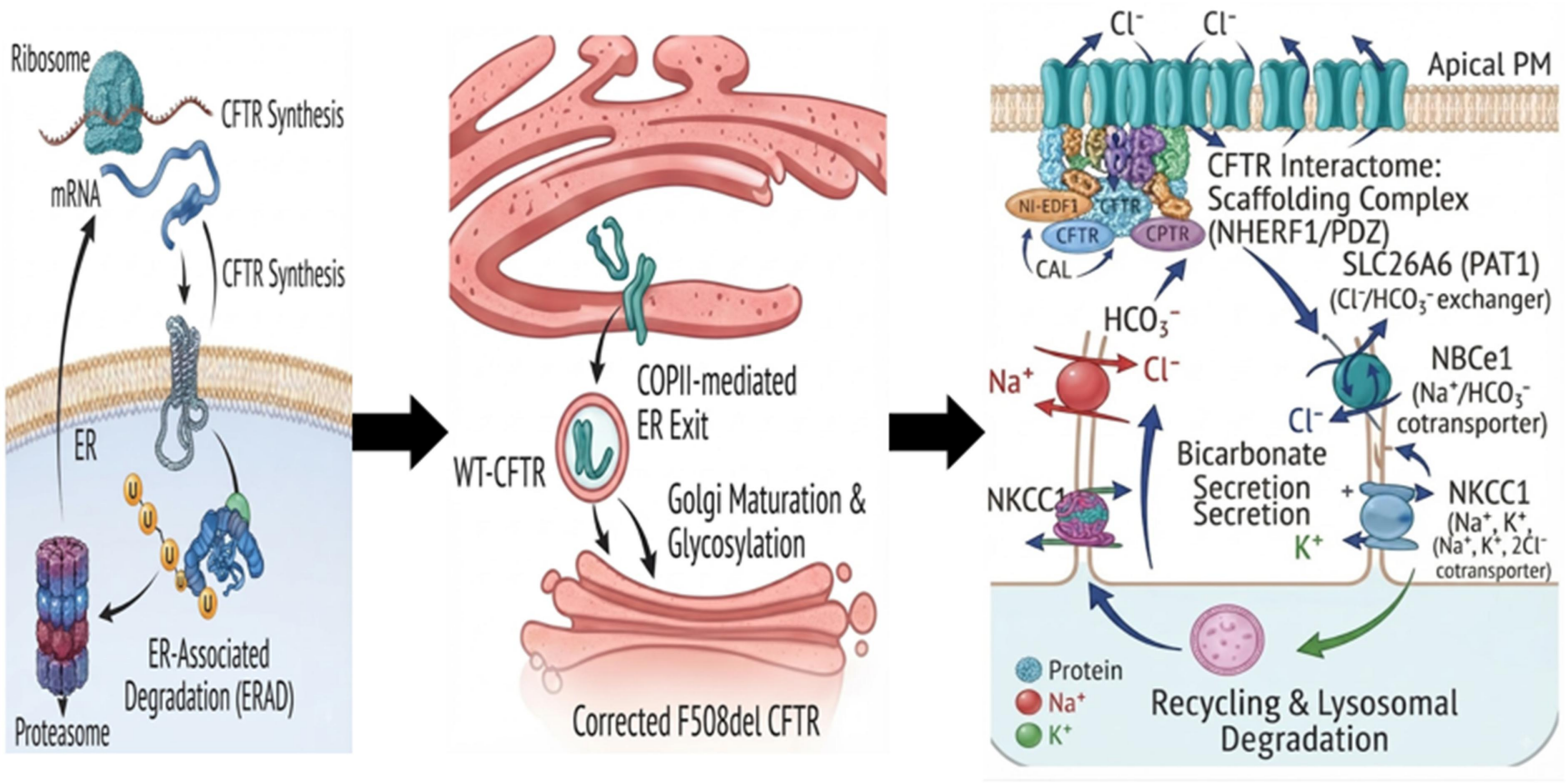
CFTR pathway and pancreatic ductal function.

Accumulating evidence suggested a key mechanism for controlling the quantity and quality of CFTR. The ER is where CFTR is made and folded, and ER quality-control systems continuously evaluate its structure there [48]. Chaperone proteins like Hsp70, Hsp90, and calnexin identify misfolded CFTR and subsequently attract E3 ubiquitin ligases like CHIP, RMA1 (RNF5), and gp78 [49]. These ligases designate the faulty CFTR for destruction by attaching ubiquitin chains to its lysine residues [48]. The tagged CFTR is then broken down by the 26S proteasome in the cytosol after being extracted from the ER membrane by the ERAD pathway, which involves the ATPase p97/VCP [50]. Fbxo45 promotes pancreatic tumorigenesis by enhancing NEK6-mediated ubiquitination and degradation of USP49, which may disrupt CFTR-regulated secretory processes in exocrine pancreatic cells, increasing cell proliferation and tumor growth [51]. CFTR and the resulting clinical features of cystic fibrosis are primarily driven by disruptions in ubiquitin-dependent regulatory pathways (Figure 2).

### Endocytic recycling and membrane

Suggestions from previous studies have demonstrated that a highly regulated quality control system in pancreatic ductal epithelial cells determines whether CFTR channels are degraded, recycled, or retained at the apical membrane to support chloride and bicarbonate secretion [52]. These pathways direct defective CFTR into multivesicular bodies that mature into late endosomes and fuse with lysosomes, resulting in channel degradation [53]. In cystic fibrosis, increased ubiquitination and impaired recycling shorten the surface half-life of CFTR, reducing bicarbonate-rich fluid secretion. Consequently, defective endocytic recycling and enhanced lysosomal targeting worsen pancreatic ductal dysfunction, contributing to ductal obstruction, inflammation, and exocrine pancreatic disease [54].

### Regulatory interactors modulating CFTR function

Previous studies demonstrated that regulatory interactors modulate CFTR function. Combined exposure to IL-1β, IL-6, IFN-γ, and TNF-α significantly enhanced CFTR expression and CFTR-dependent anion (primarily bicarbonate) secretion in pancreatic ductal epithelial monolayers, while individual cytokines had minimal effects, and epithelial barrier function remained largely intact [55]. Excessive secretagogue-induced ER stress activates the PERK–CHOP pathway, impairing CFTR folding and trafficking, thereby exacerbating CFTR channelopathy and promoting enzyme retention and pancreatic injury [56]. Failure of these integrated interactions results in the dehydration and acidification of pancreatic juice, leading to protein precipitation, ductal obstruction, and the progressive fibro-inflammatory damage characteristic of cystic fibrosis-associated pancreatic insufficiency.

## Discussion

### CFTR dysfunction and apical protein targeting

In the exocrine pancreas, CFTR dysfunction extends beyond impaired chloride and bicarbonate conductance to directly disrupt apical protein targeting and epithelial polarity, highlighting a broader role in maintaining epithelial organization [57]. Dysfunction of this channel, resulting from mutations in the *CFTR* gene, causes cystic fibrosis [58]. Accumulating evidence indicates that, in pancreatic ductal epithelia, CFTR apical localization depends on coordinated ER–Golgi trafficking and interactions with adaptor complexes and PDZ-domain scaffold proteins such as NHERF1 and NHERF2 [59]. Channelopathic CFTR mutations, particularly Class II (e.g. F508del) and Class III variants, compromise folding and ER exit, thereby shifting CFTR toward proteasomal degradation or non-apical mislocalization and reducing functional apical channel density [39]. This defective sorting reduces bicarbonate-rich fluid secretion, a key determinant of ductal luminal pH, enzyme solubility, and protection against intrapancreatic enzyme activation [39]. However, whether these trafficking defects arise primarily from direct loss of CFTR–scaffold interactions or secondarily from altered ionic homeostasis remains unresolved. The loss of CFTR from the apical membrane also destabilizes multiprotein complexes that organize other transporters, including SLC26 anion exchangers and aquaporins [60]. Thus, CFTR channelopathies indirectly propagate a broader trafficking defect, reinforcing the concept that CFTR functions as both an ion channel and a trafficking hub in exocrine pancreatic epithelia.

### Intersection with ER stress and the unfolded protein response

Protein sorting defects caused by misfolded CFTR impose a chronic burden on the ER quality-control machinery, a stress that is particularly consequential in the exocrine pancreas due to its high secretory demand [61]. Sustained activation of the unfolded protein response (UPR) in ductal cells shifts the balance from adaptive folding toward terminal ER stress, reducing secretory capacity and sensitizing cells to dysfunction [62]. In the exocrine pancreas, where secretory throughput is exceptionally high, this stress environment can compromise the trafficking and maturation of digestive enzyme precursors in acinar cells, likely through altered ductal signaling and luminal conditions that disrupt duct–acinar cross-talk [63,64]. Furthermore, aberrant CFTR retention in the ER may sequester molecular chaperones and COPII components, creating competition for limited trafficking resources and indirectly impairing the export of unrelated secretory proteins [65]. This mechanism provides a direct link between CFTR channelopathies, chronic ER stress, and the broader secretory insufficiency that characterizes cystic fibrosis–related pancreatic disease [66,67].

### Golgi and endosomal sorting defects

Beyond ER exit, CFTR mutations perturb post-Golgi sorting and recycling pathways, extending trafficking defects to later stages that are critical for maintaining apical membrane composition in pancreatic ductal epithelia [49]. Defective interactions with Rab GTPases, SNARE proteins, and clathrin adaptor complexes disrupt endosome-to-apical recycling, impairing the return of CFTR to the apical membrane after internalization [68]. In pancreatic duct cells, impaired recycling reduces apical CFTR surface stability even when partial channel function is preserved, limiting the functional benefit of residual or modulator-corrected CFTR [69]. Endosomal trafficking defects can additionally redirect other apical proteins toward lysosomal degradation, amplifying the loss of epithelial polarity [70]. These polarity defects alter ductal architecture and impair luminal flushing, creating a microenvironment that favors protein precipitation and ductal obstruction, hallmark features of pancreatic pathology in CFTR-related disorders [71].

### Functional consequences for exocrine pancreatic physiology

The convergence of impaired ion transport and defective protein sorting in CFTR channelopathies has profound consequences for exocrine pancreatic function [72,73]. Reduced bicarbonate secretion, compounded by mislocalized transporters and enzymes due to defective protein sorting, lowers intraductal pH and favors premature activation and aggregation of digestive enzymes [57]. Altered trafficking of zymogen granule components and membrane transporters further disrupts coordinated enzyme secretion, increasing acinar stress and susceptibility to injury [74]. Even CFTR mutations associated with “pancreatic sufficiency” can cause subtle trafficking defects, predisposing individuals to recurrent pancreatitis and highlighting protein sorting as a modifier of disease severity beyond channel conductance. This underscores the importance of protein sorting defects as modifiers of disease severity beyond classical channel conductance measurements [75].

### Therapeutic implications and future directions

The expanding recognition that CFTR channelopathies encompass disruptions in protein sorting and trafficking machinery reframes CF not solely as a channel-gating disorder but as a disease of spatial proteostasis [76]. CFTR modulators, especially the triple combination of ivacaftor, tezacaftor, and elexacaftor, improved pancreatic epithelial cell function in F508del-CFTR cells and remained effective under inflammatory conditions, although they did not reduce inflammation-related cytokine production. Although CFTR modulators, including correctors and potentiators, partially rescue protein folding and channel activity, their therapeutic ceiling is likely imposed by persistent defects in CFTR stabilization, polarized targeting, and recycling [75]. Highly effective modulator therapies (HEMTs), particularly the triple combination therapy ETI (elaxacaftor-tezacaftor-ivacaftor), have shown beneficial effects on pancreatic sufficiency (restoration of normal digestive enzyme production) [77–79], pancreatic stiffness and structural stress [80], and even on the glucose homeostatic function of the endocrine pancreas [81,82], although larger and longer-term studies, especially in children, are needed to confirm these effects. Accordingly, strategies that target auxiliary regulatory pathways such as reinforcement of endoplasmic reticulum proteostasis, stabilization of PDZ domain–dependent scaffolding complexes, and modulation of endosomal trafficking and recycling circuits represent a rational next generation of combinatorial therapies. These approaches may be particularly impactful in the exocrine pancreas, where precise subcellular localization of CFTR is essential for ductal fluid and bicarbonate secretion. Future investigations should leverage high-resolution imaging, spatial proteomics, and systems-level interactome analyses to define CFTR-centered trafficking networks in pancreatic epithelial cells. Dissecting how disease-causing CFTR variants rewire intracellular sorting pathways may identify novel biomarkers of pancreatic dysfunction and uncover intervention points that extend beyond channel correction, with the potential to preserve exocrine pancreatic architecture and function in CFTR-related disorders.

### Strengths and limitations

#### Strengths

This systematic review’s integrative methodological approach, which combines narrative synthesis with PRISMA-compliant systematic review techniques, is one of its main strengths. This approach maintained scientific rigor in research identification, selection, and data extraction while enabling a thorough assessment of the literature on CFTR channelopathies. The review covers both fundamental and new insights into CFTR sorting and trafficking abnormalities in pancreatic ductal epithelial cells by covering a broad temporal and experimental scope.

The analysis’ mechanistic depth is another important strength. This study highlights the crucial importance of protein sorting machinery, including ER quality control systems, Golgi processing, endocytic recycling pathways, and ubiquitin-dependent degradation mechanisms, rather than concentrating only on CFTR ion transport abnormalities. This more comprehensive view expands conventional classifications of channel failure in the exocrine pancreas by redefining CFTR channelopathies as disorders of membrane trafficking and proteostasis.

The incorporation of several experimental models, including human pancreatic tissue, patient-derived epithelial cells, genetically modified animal models, and in vitro trafficking systems, further enhances the review. By connecting molecular sorting errors to physiological consequences such as reduced bicarbonate secretion, ductal blockage, and exocrine pancreatic insufficiency, this diversity enhances the validity of the conclusions. Translational relevance is increased by the convergence of cellular, pathophysiological, and genetic findings.

Lastly, a clear and organized framework is provided by the thematic categorization of data into several stages of CFTR life-cycle regulation: biosynthesis and folding, intracellular trafficking, endocytic sorting, recycling versus lysosomal destruction, and apical membrane stability. In addition to elucidating how CFTR dysfunction is exacerbated by protein sorting errors, this structure identifies possible therapeutic intervention points, such as improving CFTR surface stability in the exocrine pancreas and modifying trafficking pathways.

### Limitations

Despite this review’s advantages, a few drawbacks should be noted. First, there are still a few studies that specifically look at the protein sorting mechanism in CFTR channelopathies in the exocrine pancreas, which makes it difficult to derive definitive mechanistic insights. Direct comparison of results is complicated by the heterogeneity of the included investigations, which include genetic analysis, in vitro epithelial models, animal studies, and pharmaceutical interventions. Second, the reliance on experimental models may not entirely reflect human pancreatic physiology or the complex pathophysiology of CFTR-related pancreatic disease, particularly regarding duct–acinar interactions, luminal flow, and proteostasis under high secretory load. Thirdly, language and publishing bias may have been created by excluding non-English articles and full texts that were unavailable, thus omitting pertinent data from other areas or new research. Lastly, there is little clinical data in human patients, despite the review highlighting protein sorting machinery as a possible therapeutic target for restoring CFTR function and enhancing pancreatic output. As a result, the translational usefulness of altering these pathways is still mainly hypothetical and needs more confirmation in clinical research and models derived from patients.

### Conclusions and recommendations

#### Conclusions

CFTR folding, maturation, and trafficking defects are well-established contributors to CFTR loss of function and play a central role in the pathogenesis of cystic fibrosis. Disruptions in ER quality control, ERAD, Golgi processing, and vesicular trafficking can impair CFTR delivery to the apical membrane, reducing channel activity and altering epithelial fluid and bicarbonate secretion. Although the relevance of these proteostatic and trafficking abnormalities to exocrine pancreatic disease is biologically plausible, direct evidence linking specific defects in CFTR sorting machinery to pancreatic dysfunction remains insufficient and requires more clinical investigations. Future studies employing pancreatic ductal cell models, patient-derived systems, organoids, and functional assays are needed to define the contribution of CFTR trafficking pathways to pancreatic disease and to evaluate whether targeting these pathways can provide effective therapeutic benefits beyond current treatments.

### Recommendations

In-depth mechanistic analyses of how particular protein sorting machinery, such as Rab GTPases, COPII/COPI components, adaptor protein complexes, SNAREs, and tethering factors, control CFTR folding, trafficking, and apical membrane stability in pancreatic ductal cells under both physiological and pathological conditions should be the main focus of future research. Examining how ER quality control, ERAD, and Golgi sorting interact to determine CFTR fate and ductal secretory capacity should receive special attention. To enable early diagnosis of exocrine pancreatic dysfunction and classification of disease severity, particularly in cystic fibrosis-related and idiopathic pancreatitis, efforts should also concentrate on finding reliable molecular and imaging biomarkers of CFTR misfolding and mislocalization. Translational research is required to assess therapeutic approaches that target intracellular trafficking pathways and proteostasis, such as folding modulators, trafficking enhancers, and ER stress-attenuating agents, as disease-modifying substitutes or supplements to enzyme replacement therapy. Bridging experimental findings with clinical significance will require longitudinal human investigations that include genetics, cell biology, and functional pancreatic outcomes. Lastly, further research into understudied regulatory layers, such as the exocrine pancreas’ circadian regulation of protein sorting machinery, inflammatory signaling, and metabolic stress responses, may reveal new therapeutic windows and enable more accurate, patient-specific treatment of pancreatic channelopathies linked to CFTR.

## Supplementary Material

Revised Supplementary File S2.docx

Revised Supplementary File S3.docx

Revised Supplementary File S1.docx

Revised Supplementary File S4.docx

## Data Availability

The data used to synthesize information in this review article are available as Supplementary Files S1 to S4 submitted together with this article.
