# Supplementary material for: Protein sorting and proteostasis mechanisms in CFTR-related exocrine pancreas dysfunction: A systematic narrative review
Source: Channels (Austin). 2026 Sep 12;20(1):2731660. doi: 10.1080/19336950.2026.2731660 (PMC13577266; doi:10.1080/19336950.2026.2731660)
Supplement: Supplementary file 1 [file KCHL_A_2731660_SM0083.docx]

**Supplementary File S4: Risk of Bias Assessment Report**

| **Author(s) and Year** | **Study Design / Selection Bias** | **Measurement Bias** | **Confounding** | **Reporting Bias** | **Other Bias** | **Overall Risk of Bias** |
| --- | --- | --- | --- | --- | --- | --- |
| Petek Ballar et al., 2010 | Moderate: human cell culture, not representative of whole organism | Low: validated assays (Western blot, RNAi, co-IP) | Moderate: ERAD complexity, multiple ligases | Low: clear reporting of gp78/Hrd1 roles | Possible cell line-specific effects | Moderate |
| Hocevar et al., 2020 | Low: controlled in vitro acinar cell model | Low: multiple assays (MTT, qPCR, WB) | Moderate: toxicant exposure overlaps with stressors | Low: detailed methodology | Environmental toxicant dose relevance | Moderate |
| Ludovico & Baroni, 2025 | Moderate: pancreatic epithelial cell line only | Low: electrophysiology and immunofluorescence robust | Moderate: cytokine effects not fully controlled | Low: transparent reporting | Industry involvement in CFTR modulators | Moderate |
| Riepe et al., 2024 | Moderate: human cell lines, limited generalizability | Low: multiple assays confirm findings | Moderate: ERAD ligases RNF5/RNF185 may interact with other pathways | Low: clear reporting | Corrector compounds may have off-target effects | Moderate |
| Navis & Bagnat, 2015 | Low: zebrafish genetic model strong for developmental biology | Low: histology and functional assays reliable | Moderate: extrapolation to humans | Low: well reported | Zebrafish developmental differences vs humans | Moderate |
| Gnann et al., 2024 | Moderate: yeast model less representative of human pancreas | Low: genetic mutants and biochemical assays robust | High: yeast ERAD differs from mammalian | Low: clear reporting | Yeast ERAD differs substantially from mammalian | Moderate |
| Kostenko et al., 2018 | Moderate: mouse acinar cells, ex vivo/in vivo | Low: multiple imaging and secretion assays | Moderate: c-Src signaling overlaps with other kinases | Low: transparent | Src kinase redundancy complicates interpretation | Moderate |
| Lee & Goldberg, 2010 | Low: purified protein complexes, structural biology | Low: crystallography precise | Low: minimal confounding | Low: clear reporting | Structural biology may not reflect dynamic physiology | Low |
| Rizzo et al., 2021 | Moderate: HeLa cells, not pancreatic | Low: advanced imaging and lipidomics | Moderate: GOLPH3 effects vary by cell type | Low: detailed | HeLa cells not pancreas-specific | Moderate |
| Etienne et al., 2005 | Moderate: CFPAC-1 cell line | Low: EM and immunocytochemistry robust | Moderate: transfection may introduce artifacts | Low: clear | Transfection artifacts possible | Moderate |
| Dahan et al., 2005 | Moderate: rodent acinar cells | Low: live-cell imaging reliable | Moderate: CCK stimulation overlaps with other pathways | Low: clear | Ex vivo stimulation may not mimic in vivo | Moderate |
| Younger et al., 2006 | Moderate: HEK293 cells | Low: multiple QC assays | Moderate: ERAD complexity | Low: clear | HEK293 cells not pancreas-specific | Moderate |
| Tsukasa et al., 2010 | Moderate: HeLa/IB3 cells | Low: siRNA and ubiquitination assays robust | Moderate: peripheral QC overlaps with ERAD | Low: clear | Peripheral QC may differ in vivo | Moderate |
| Jarosch et al., 2002 | Moderate: yeast ERAD model | Low: genetic and biochemical assays strong | Moderate: yeast vs mammalian differences | Low: clear | Yeast ERAD extrapolation limits | Moderate |
| Linhui et al., 2022 | Moderate: pancreatic cancer lines + xenografts | Low: multiple assays robust | Moderate: tumor heterogeneity | Low: clear | Cancer heterogeneity and xenograft variability | Moderate |
| Heda et al., 2001 | Moderate: MDCK epithelial cells | Low: pulse-chase reliable | Moderate: epithelial model not fully pancreatic | Low: clear | MDCK cells not pancreatic | Moderate |
| Sharma et al., 2004 | Moderate: epithelial cell lines | Low: confocal microscopy robust | Moderate: recycling vs degradation pathways overlap | Low: clear | Temperature rescue not physiologic | Moderate |
| Bomberger et al., 2009 | Moderate: airway epithelial cells | Low: proteomic and functional assays robust | Moderate: airway vs pancreatic differences | Low: clear | Airway vs pancreatic differences | Moderate |
| Angyal et al., 2024 | Moderate: porcine pancreatic organoids | Low: electrophysiology robust | Moderate: cytokine interactions complex | Low: clear | Porcine organoids differ from human | Moderate |
| Constanze et al., 2007 | Moderate: rat acinar cells | Low: UPR markers reliable | Moderate: secretagogue overlap | Low: clear | Secretagogue doses may exceed physiologic | Moderate |
