# Supplementary material for: Protein sorting and proteostasis mechanisms in CFTR-related exocrine pancreas dysfunction: A systematic narrative review
Source: Channels (Austin). 2026 Sep 12;20(1):2731660. doi: 10.1080/19336950.2026.2731660 (PMC13577266; doi:10.1080/19336950.2026.2731660)
Supplement: Supplementary file 2 [file KCHL_A_2731660_SM0084.docx]

| **Section and Topic** | **Item #** | **Checklist item** | **Location where item is reported** |
| --- | --- | --- | --- |
| **TITLE** | | |  |
| Title | 1 | Identify the report as a systematic review. | **Protein sorting and proteostasis mechanisms in CFTR-related exocrine pancreas dysfunction: a systematic narrative review** |
| **ABSTRACT** | | |  |
| Abstract | 2 | See the PRISMA 2020 for Abstracts checklist. | **Background:** The pancreas consists of exocrine and endocrine compartments. In the exocrine pancreas, cystic fibrosis transmembrane conductance regulator (CFTR) functions mainly in ductal epithelial cells as a chloride and bicarbonate channel. Its activity depends on proper protein folding, trafficking, and localization to the apical membrane.  **Objectives:** This systematic narrative review aims to synthesize the available evidence on the role of protein sorting machinery in CFTR channelopathies and its contribution to exocrine pancreatic dysfunction.  **Methodology:** A thorough search was conducted using PRISMA criteria on PubMed, Wiley Online Library, and Scopus for studies published in English between January 2000 and November 2025.  **Results:** Twenty studies that met the inclusion criteria were included in this review**.** Pathogenic CFTR variants impair protein folding, endoplasmic reticulum (ER) exit, and endosomal recycling, resulting in reduced apical membrane expression and stability. These defects disrupt the localization of associated transporters and secretory proteins, impair ductal bicarbonate secretion, alter zymogen handling, and promote acinar injury, although these claims are supported mainly by indirect experimental models and therefore require clinical confirmation.  **Conclusion:** CFTR channelopathies in the exocrine pancreas encompass both ion transport defects and broader disruptions of protein sorting machinery. CFTR may contribute to the assembly, stabilization, or localization of selected apical transport complexes, and its loss can secondarily alter epithelial organization. Therapeutic approaches targeting both channel correction and intracellular trafficking may improve pancreatic function and mitigate disease progression.  **Keywords:** CFTR, Channelopathies, Exocrine pancreas, Protein trafficking |
| **INTRODUCTION** | | |  |
| Rationale | 3 | Describe the rationale for the review in the context of existing knowledge. | Despite extensive research on the role of the cystic fibrosis transmembrane conductance regulator (CFTR) as a chloride and bicarbonate channel, most existing studies have primarily focused on its ion transport function in the pathogenesis of cystic fibrosis. However, emerging evidence indicates that CFTR dysfunction is also closely linked to abnormalities in intracellular protein folding, trafficking, and apical membrane targeting, processes governed by complex protein sorting machinery. Disruptions in these pathways contribute not only to defective channel activity but also to mislocalization of associated transporters and secretory proteins, particularly in the exocrine pancreas. Although individual studies have explored aspects of CFTR trafficking and its impact on pancreatic function, the evidence remains fragmented and lacks comprehensive synthesis. |
| Objectives | 4 | Provide an explicit statement of the objective(s) or question(s) the review addresses. | **Objective:**  This review focuses on how protein sorting machinery abnormalities contribute to CFTR channelopathies in the exocrine pancreas by examining the roles of molecular chaperones, ubiquitin ligases, deubiquitinating enzymes, and vesicular trafficking systems in CFTR maturation and stability, highlighting the mechanistic links between intracellular sorting defects and pancreatic pathology.  **Research Question:**  What is the role of intracellular protein sorting mechanisms in regulating CFTR trafficking and function, and how do defects in these processes contribute to exocrine pancreatic dysfunction in CFTR-related disease? |
| **METHODS** | | |  |
| Eligibility criteria | 5 | Specify the inclusion and exclusion criteria for the review and how studies were grouped for the syntheses. | **Inclusion Criteria:**   - Studies investigating the cystic fibrosis transmembrane conductance regulator (CFTR) in the exocrine pancreas. - Studies examining intracellular protein sorting, trafficking, folding, or apical membrane localization of CFTR. - Experimental, observational, or clinical studies that assess the impact of CFTR dysfunction on pancreatic exocrine function. - Publications in English, from January 2000 to November 2025.   **Exclusion Criteria:**   - Studies not focused on CFTR or pancreatic exocrine function. - Reviews, commentaries, conference abstracts, or editorials without primary data. - Non-English publications. - Studies focusing exclusively on pulmonary CFTR function or non-pancreatic tissues. |
| Information sources | 6 | Specify all databases, registers, websites, organisations, reference lists and other sources searched or consulted to identify studies. Specify the date when each source was last searched or consulted. | All articles retrieved from Wiley Online Library, PubMed and Scopus were manually assessed based on their titles, abstracts, and keywords. During the eligibility assessment phase, full-text articles were thoroughly examined for inclusion. Two independent reviewers carried out the screening process, and any discrepancies or uncertainties were resolved through discussion with a third author. |
| Search strategy | 7 | Present the full search strategies for all databases, registers and websites, including any filters and limits used. | A comprehensive literature search was performed using the Wiley Online Library, PubMed, and Scopus, covering studies published from January 2000 to November 2025. The search targeted research investigating CFTR channelopathies caused by abnormalities in the protein sorting and trafficking machinery within the exocrine pancreas. The search strategy combined controlled vocabulary and free-text keywords. Core terms related to CFTR dysfunction included: “CFTR,” “CFTR mutations,” “CFTR channelopathies,” “cystic fibrosis transmembrane conductance regulator,” “CFTR misfolding,” and “CFTR trafficking defects.” To capture the role of intracellular sorting systems, we used protein-processing terms such as “protein sorting,” “protein trafficking,” “endoplasmic reticulum,” “Golgi apparatus,” “ER-associated degradation (ERAD),” “chaperone proteins,” “COPII vesicles,” “quality control machinery,” and “ubiquitin-proteasome pathway.” Because the focus is on the exocrine pancreas, organ-specific keywords were included: “exocrine pancreas,” “pancreatic acinar cells,” “pancreatic ductal cells,” “pancreatic secretion,” and “bicarbonate secretion.” To ensure broad coverage of experimental approaches, additional search terms were incorporated for study models such as “animal models,” “mouse models,” “rat models,” “organoids,” “cell lines,” and “*in vitro* studies.” |
| Selection process | 8 | Specify the methods used to decide whether a study met the inclusion criteria of the review, including how many reviewers screened each record and each report retrieved, whether they worked independently, and if applicable, details of automation tools used in the process. | Two authors independently screened all titles, abstracts, and full-text articles against the predefined inclusion and exclusion criteria, with disagreements resolved through discussion with a third author. No automation tools were used during any stage of the screening or eligibility assessment process. |
| Data collection process | 9 | Specify the methods used to collect data from reports, including how many reviewers collected data from each report, whether they worked independently, any processes for obtaining or confirming data from study investigators, and if applicable, details of automation tools used in the process. | Data were extracted independently by two authors using a standardized, pre-defined data collection form, and any discrepancies were resolved through consensus discussion among all authors. No automation tools were used, and no additional data were sought from study investigators. |
| Data items | 10a | List and define all outcomes for which data were sought. Specify whether all results that were compatible with each outcome domain in each study were sought (e.g. for all measures, time points, analyses), and if not, the methods used to decide which results to collect. | **The primary outcomes included :**   - ER quality control and ERAD - Golgi maturation and vesicular trafficking - Ubiquitin-mediated degradation pathways - Endocytic recycling and membrane stability - Regulatory interactors modulating CFTR function |
|  | 10b | List and define all other variables for which data were sought (e.g. participant and intervention characteristics, funding sources). Describe any assumptions made about any missing or unclear information. | Additional variables extracted included study characteristics (author, year, country), experimental model details (species, strain, cell line, sample size, sex), exposure characteristics (type of endocrine disruptor, dose, duration, administration route), target tissues assessed, and funding or conflict-of-interest statements where reported. When information was missing or unclear, we recorded it as “not reported” and made no assumptions unless the study explicitly indicated standard laboratory procedures, in which case common methodological conventions were noted but not treated as confirmed data. |
| Study risk of bias assessment | 11 | Specify the methods used to assess risk of bias in the included studies, including details of the tool(s) used, how many reviewers assessed each study and whether they worked independently, and if applicable, details of automation tools used in the process. | Risk of bias in the included studies was assessed using structured appraisal tools appropriate for each study type: Systematic Review Centre for Laboratory Animal Experimentation (SYRCLE)’s Risk of Bias tool for animal studies^32^, human studies were assessed using the Cochrane RoB 1 tool, while in vitro studies were assessed using the modified Checklist for Reporting In Vitro Studies (CRIS), emphasizing experimental design, reproducibility, control conditions, and statistical rigor. |
| Effect measures | 12 | Specify for each outcome the effect measure(s) (e.g. risk ratio, mean difference) used in the synthesis or presentation of results. | Results were synthesized **narratively,** focusing on key outcomes related to CFTR trafficking and exocrine pancreatic function. Studies were grouped according to the type of intracellular defect (e.g., protein folding, ER exit, endosomal recycling, or apical membrane localization). For each group, reported effects on pancreatic ductal and acinar function—such as bicarbonate secretion, zymogen processing, and transporter localization—were summarized. Qualitative assessments of pancreatic tissue morphology, histopathology, and associated cellular adaptations were also described. Where possible, relationships between the severity of CFTR trafficking defects and functional or structural pancreatic outcomes were highlighted. |
| Synthesis methods | 13a | Describe the processes used to decide which studies were eligible for each synthesis (e.g. tabulating the study intervention characteristics and comparing against the planned groups for each synthesis (item #5)). | Studies were grouped according to the type of CFTR defect or trafficking pathway investigated (protein folding, ER exit, endosomal recycling, apical membrane localization). Inclusion in a synthesis was determined by whether the study assessed CFTR trafficking mechanisms and reported outcomes related to exocrine pancreatic function, including bicarbonate secretion, zymogen handling, or associated transporter localization. |
|  | 13b | Describe any methods required to prepare the data for presentation or synthesis, such as handling of missing summary statistics, or data conversions. | Data extraction included recording the type of CFTR mutation or trafficking defect, experimental model (cell line, organoid, or animal), and measured outcomes. Where necessary, qualitative outcomes such as histopathological descriptions or protein localization were standardized for comparison across studies, and data were tabulated to facilitate narrative synthesis. |
|  | 13c | Describe any methods used to tabulate or visually display results of individual studies and syntheses. | Results were summarized in tables highlighting study characteristics, CFTR variant or defect studied, cellular pathways affected, and pancreatic outcomes. Key findings were also displayed schematically to illustrate the relationship between CFTR trafficking defects and exocrine pancreatic dysfunction. |
|  | 13d | Describe any methods used to synthesize results and provide a rationale for the choice(s). If meta-analysis was performed, describe the model(s), method(s) to identify the presence and extent of statistical heterogeneity, and software package(s) used. | Results were synthesized **narratively** due to heterogeneity in experimental models, outcomes measured, and reporting formats. Studies were grouped by type of trafficking defect to allow identification of patterns in functional and morphological consequences on the exocrine pancreas. |
|  | 13e | Describe any methods used to explore possible causes of heterogeneity among study results (e.g. subgroup analysis, meta-regression). | Potential sources of heterogeneity, including differences in CFTR variants, experimental models, and measured outcomes, were examined. Narrative comparison highlighted consistencies and discrepancies in reported functional impairments and morphological changes across studies, providing insight into the variability of exocrine pancreatic phenotypes. |
|  | 13f | Describe any sensitivity analyses conducted to assess robustness of the synthesized results. | Due to the qualitative nature of included studies and limited quantitative data, formal sensitivity analyses were not performed. The robustness of findings was evaluated by comparing results across different experimental models and study designs to ensure conclusions were not driven by single-study outcomes. |
| Reporting bias assessment | 14 | Describe any methods used to assess risk of bias due to missing results in a synthesis (arising from reporting biases). | We assessed the risk of bias due to missing results by examining selective outcome reporting within studies, comparing reported outcomes with those prespecified in protocols or Methods sections. |
| Certainty assessment | 15 | Describe any methods used to assess certainty (or confidence) in the body of evidence for an outcome. | A formal GRADE assessment was not conducted due to considerable variation in experimental models, hormonal contraceptive types, exposure durations, and measured outcomes. |
| **RESULTS** | | |  |
| Study selection | 16a | Describe the results of the search and selection process, from the number of records identified in the search to the number of studies included in the review, ideally using a flow diagram. |  |
|  | 16b | Cite studies that might appear to meet the inclusion criteria, but which were excluded, and explain why they were excluded. | In addition to CFTR variants and their effects on protein trafficking, data were extracted on study characteristics (year, country, experimental model, sample size), type of exocrine pancreatic cells or tissues studied, methods used to assess CFTR localization or function, associated protein sorting machinery components (e.g., chaperones, endocytic recycling markers), and funding sources. When information was missing or unclear, assumptions were made based on related methodological descriptions or by contacting study authors where possible. |
| Study characteristics | 17 | Cite each included study and present its characteristics. | This is available in the study characteristics table |
| Risk of bias in studies | 18 | Present assessments of risk of bias for each included study. | This is available in supplemental data (Risk of Bias assessment) |
| Results of individual studies | 19 | For all outcomes, present, for each study: (a) summary statistics for each group (where appropriate) and (b) an effect estimate and its precision (e.g. confidence/credible interval), ideally using structured tables or plots. | Due to substantial variation in study design, experimental models (cell lines, organoids, and animal models), CFTR variants studied, and measures of pancreatic exocrine function, consistent summary statistics and comparable effect estimates could not be derived. Most studies did not report standardized quantitative outcomes, such as group-level means with variability measures or effect sizes with confidence intervals. This heterogeneity precluded pooled quantitative analyses. Consequently, results of individual studies were summarized narratively, focusing on the direction and nature of CFTR trafficking defects, associated disruptions in protein sorting machinery, and their effects on pancreatic ductal and acinar function, rather than statistical comparisons between studies. |
| Results of syntheses | 20a | For each synthesis, briefly summarise the characteristics and risk of bias among contributing studies. | The synthesis included 20 studies investigating CFTR trafficking and exocrine pancreatic function. Study characteristics varied in terms of experimental model (cell lines, organoids, and animal models), CFTR variants analyzed, and outcomes measured. Risk of bias was generally moderate, with most studies clearly describing experimental procedures but lacking standardized quantitative measures. Findings were interpreted in the context of these methodological differences. |
|  | 20b | Present results of all statistical syntheses conducted. If meta-analysis was done, present for each the summary estimate and its precision (e.g. confidence/credible interval) and measures of statistical heterogeneity. If comparing groups, describe the direction of the effect. | Due to substantial heterogeneity across studies in CFTR variants, experimental systems, and outcome measures, pooled quantitative synthesis was not possible. Most studies did not report group-level means, variability measures, or standardized effect sizes. Consequently, results were presented narratively, highlighting patterns and directions of CFTR trafficking defects and their impact on exocrine pancreatic function rather than statistical effect estimates. |
|  | 20c | Present results of all investigations of possible causes of heterogeneity among study results. | Differences in CFTR variants, experimental models, and outcomes were explored to explain variability among study results. Narrative comparison revealed that protein folding defects consistently impaired ER exit and apical membrane localization, while endosomal recycling defects mainly disrupted transporter trafficking. Variability in morphological and functional outcomes reflected differences in model systems and CFTR defects. |
|  | 20d | Present results of all sensitivity analyses conducted to assess the robustness of the synthesized results. | Formal sensitivity analyses were not conducted due to the qualitative nature of the included studies and lack of standardized quantitative data. The robustness of synthesized conclusions was assessed by comparing findings across multiple experimental models and CFTR variants, ensuring that reported trends were not driven by individual studies. |
| Reporting biases | 21 | Present assessments of risk of bias due to missing results (arising from reporting biases) for each synthesis assessed. | Due to the heterogeneity of included studies and the lack of standardized quantitative outcomes, formal assessment of reporting bias (e.g., publication bias or selective outcome reporting) was not feasible. However, potential bias was considered qualitatively: studies reporting positive or novel CFTR trafficking defects were more frequently published, whereas negative or null findings were less represented. This potential reporting bias was taken into account when interpreting synthesized results. |
| Certainty of evidence | 22 | Present assessments of certainty (or confidence) in the body of evidence for each outcome assessed. | The overall certainty of evidence was considered moderate to low, reflecting the diversity of experimental models, CFTR variants, and outcomes assessed. Most studies provided mechanistic insights but lacked standardized quantitative measurements, limiting the ability to draw strong conclusions. Narrative synthesis was used to highlight consistent trends across studies, while acknowledging uncertainty arising from methodological variability and limited reproducibility between models. |
| **DISCUSSION** | | |  |
| Discussion | 23a | Provide a general interpretation of the results in the context of other evidence. | This review demonstrates that CFTR channelopathies in the exocrine pancreas involve not only impaired chloride and bicarbonate transport but also widespread disruptions of intracellular protein sorting machinery. Findings from the included studies consistently indicate that defects in protein folding, ER exit, and endosomal recycling lead to reduced apical membrane localization of CFTR and associated transporters. These trafficking abnormalities contribute to impaired ductal bicarbonate secretion, altered zymogen processing, and acinar injury, complementing previous knowledge about CFTR’s ion transport function and expanding understanding of its dual role in pancreatic physiology. |
|  | 23b | Discuss any limitations of the evidence included in the review. | The evidence base was limited by substantial heterogeneity across studies, including differences in CFTR variants, experimental models (cell lines, organoids, and animal models), and outcome measures. Most studies lacked standardized quantitative reporting, such as effect sizes or variability measures, which prevented meta-analysis. Additionally, risk of bias was moderate, with selective reporting and underrepresentation of negative results potentially influencing conclusions. These limitations reduce the certainty of evidence and the ability to generalize findings to human pancreatic disease. |
|  | 23c | Discuss any limitations of the review processes used. | Limitations of this review include restricting the search to English-language publications and three databases (PubMed, Wiley Online Library, and Scopus), which may have excluded relevant studies. Narrative synthesis was used due to heterogeneity, which, while appropriate, does not provide quantitative estimates of effect. Furthermore, decisions regarding study grouping and outcome interpretation were qualitative, potentially introducing subjectivity in the synthesis process. |
|  | 23d | Discuss implications of the results for practice, policy, and future research. | The findings highlight the importance of targeting both CFTR channel function and intracellular trafficking pathways in therapeutic strategies for exocrine pancreatic dysfunction. Clinically, interventions that improve CFTR folding and trafficking could complement existing modulators focused on channel gating or stability. For future research, standardized reporting of trafficking defects, functional outcomes, and morphological assessments in comparable experimental models is recommended. Further studies integrating human pancreatic tissue or organoid systems with mechanistic analyses may clarify the contribution of trafficking defects to disease severity and variability. |
| **OTHER INFORMATION** | | |  |
| Registration and protocol | 24a | Provide registration information for the review, including register name and registration number, or state that the review was not registered. | The review was not registered in any formal systematic review registry, such as PROSPERO (International Prospective Register of Systematic Reviews) |
|  | 24b | Indicate where the review protocol can be accessed, or state that a protocol was not prepared. | A formal protocol was not publicly archived before the review began; however, a detailed internal methodology was developed and can be obtained from the corresponding author if needed. |
|  | 24c | Describe and explain any amendments to information provided at registration or in the protocol. | The review objectives remained unchanged throughout the process. Minor updates to keyword combinations and database filters were implemented after initial testing to ensure comprehensive coverage of thyroid disruptor studies, without altering the established eligibility criteria. |
| Support | 25 | Describe sources of financial or non-financial support for the review, and the role of the funders or sponsors in the review. | The review did not receive any specific financial support or funding from external organizations. No sponsors or funders were involved in the design, conduct, analysis, interpretation, or reporting of the review, and all decisions were made independently by the authors. |
| Competing interests | 26 | Declare any competing interests of review authors. | The authors report no conflicts of interest in relation to the content, analysis, or conclusions of this review. |
| Availability of data, code and other materials | 27 | Report which of the following are publicly available and where they can be found: template data collection forms; data extracted from included studies; data used for all analyses; analytic code; any other materials used in the review. | - Template used for data extraction. - Complete dataset of study characteristics and outcome measures. - Full-text PDFs of included studies, in compliance with copyright regulations. - Individual study risk-of-bias assessments using SYRCLE’s RoB tool and Cochrane RoB |

*From:*  Page MJ, McKenzie JE, Bossuyt PM, Boutron I, Hoffmann TC, Mulrow CD, et al. The PRISMA 2020 statement: an updated guideline for reporting systematic reviews. BMJ 2021;372:n7. doi: 10.1136/bmj.n71. This work is licensed under CC BY 4.0. To view a copy of this license, visit <https://creativecommons.org/licenses/by/4.0/>
