# Supplementary material for: Protein sorting and proteostasis mechanisms in CFTR-related exocrine pancreas dysfunction: A systematic narrative review
Source: Channels (Austin). 2026 Sep 12;20(1):2731660. doi: 10.1080/19336950.2026.2731660 (PMC13577266; doi:10.1080/19336950.2026.2731660)
Supplement: Supplementary file 3 [file KCHL_A_2731660_SM0085.docx]

| **Categories** | **Authors** | **Years** | **Experimental Model** | **Medium** | **Sample Size** | **Sorting Protein Investigated** | **Type of experimental intervention** | **Method Used in Assay** | **Key findings** | **DOI** |
| --- | --- | --- | --- | --- | --- | --- | --- | --- | --- | --- |
| ER quality control and ERAD | Petek Ballar et al. | 2010 | Human cell culture expressing mutant CFTR (ΔF508) | In-vitro cultured mammalian cells | Not explicitly stated | gp78, Hrd1, p97/VCP | Gene modulation (overexpression/siRNA knockdown) combined with ubiquitination and proteasomal degradation assays in cell-based models | Western blot, RNAi knockdown, co-immunoprecipitation, ubiquitination assays | gp78 promotes ubiquitination and ERAD degradation of mutant CFTR (ΔF508). Silencing gp78 or inhibiting it with SVIP leads to accumulation of CFTRΔF508. Hrd1 regulates gp78 stability and indirectly modulates CFTR degradation | [10.1016/j.biocel.2009.10.005](https://doi.org/10.1016/j.biocel.2009.10.005) |
|  | Hocevar et al. | 2020 | Mouse pancreatic acinar cell | In vitro | Cell viability: quadruplicate wells per condition (n = 4); Gene/protein assays: triplicate measurements per experiment | UPR pathway components: PERK (p-eIF2α, ATF4, CHOP), IRE1α (XBP1s), ATF6; Antioxidant genes (Sod1, Sod2, Nqo1) | PFOA treatment (50 µg/mL) for varying time points; controls with thapsigargin, tunicamycin, ionomycin, palmitoleic acid; pretreatments with PERK/IRE1α inhibitors, chemical chaperone (4-PBA), antioxidants (NAC, Tiron), IP₃ receptor inhibitor (2-APB) | MTT assay (cell viability); Western blot (protein levels of UPR markers); qRT-PCR (UPR and antioxidant gene expression); Fluo-4 AM Ca²⁺ imaging | PFOA induces ER stress and activates the unfolded protein response in pancreatic acinar cells, implicating environmental toxicants in disruption of ER proteostasis and potentially linking ERQC dysregulation to pancreatic injury | 10.1002/jbt.22561 |
|  | Ludovico and Baroni | 2025 | CFPAC‑1 pancreatic ductal epithelial cell line expressing F508del CFTR mutation | In vitro | Not Specified | F508del CFTR protein expression (immature band B, mature band C); pro‑inflammatory cytokines IL‑6, IL‑8, IL‑1β. | Pharmacological modulation using CFTR modulators | Electrophysiology (Cl⁻ currents), Immunofluorescence, Functional assays | CFTR modulators rescued F508del CFTR function in pancreatic epithelial cells, improving chloride transport and partially correcting trafficking defects | 10.3390/life15081315 |
|  | Riepe et al. | 2024 | Human cell lines expressing F508del CFTR | In Vitro | Not Specified | CFTR‑F508del (immature “Band B”, mature “Band C”) folding states RNF5 and RNF185 (ERAD ubiquitin ligases) involvement in degradation | Small-molecule corrector treatment | Western blot, Pulse-chase assays, Immunofluorescence, ERAD assays | Small-molecule correctors stabilize sequential CFTR folding states, reducing ER-associated degradation (ERAD) and enhancing trafficking to the plasma membrane | 10.1091/mbc.E23‑08‑0336 |
|  | Navis A and Bagnat M | 2015 | Zebrafish (*Danio rerio*) larval model with a cftr mutant line (cftr^pd1049/pd1049) — loss‑of‑function genetic model. | Zebrafish maintained at ~28 °C in standard fish water; mutants identified via loss of Kupffer’s vesicle lumen inflation. | Not Specified | CFTR | Genetic knockout / loss-of-function | Histology, Immunofluorescence, Functional assays (pancreatic exocrine function) | Loss of CFTR function causes severe pancreatic destruction in zebrafish larvae, highlighting the critical role of CFTR in exocrine pancreas development and maintenance | 10.1016/j.ydbio.2014.12.034 |
|  | Gnann et al., | 2024 | Saccharomyces cerevisiae (yeast) expressing CFTR | Laboratory culture conditions (yeast media) | Not explicitly numerical | Htm1p/EDEM; Cdc48p complex; Der3p/Hrd1p; Doa10p | Gene deletions and expression of CFTR; perturbation of ERAD components | Western blotting; pulse‑chase analysis; immunoprecipitation; genetic mutants | #NAME? | 10.1091/mbc.E04‑01‑0024 |
| Golgi Maturation and Vesicular Trafficking | Kostenko *et al.* | 2018 | Mouse pancreatic acinar cells (primary acini and genetically manipulated mouse models); ex vivo and in vivo analyses | Exocrine pancreas; pancreatic acinar cells with focus on the Golgi apparatus and secretory pathway | Not Specified | c-Src (Src family tyrosine kinase); Golgi and vesicular trafficking components regulating secretory cargo transit | Genetic and pharmacological modulation of c-Src activity (Src activation/inhibition); stimulation of regulated secretion | Immunofluorescence and confocal microscopy, Golgi trafficking assays, protein secretion assays, Western blotting, electron microscopy, and Src signaling manipulation | c-Src kinase regulates cargo transit through the Golgi apparatus in pancreatic acinar cells; dysregulation of this pathway alters secretory trafficking and may contribute to pancreatic pathology | DOI:10.1038/s41598-018-30370-4 |
|  | Changwook Lee and Jonathan Goldberg | 2010 | Purified protein complexes from *Bos taurus* (bovine) and subcomplexes expressed in insect cells (baculovirus system) and *E. coli* for crystallography. This is a biochemical/structural model, not a cell‑based or animal pathology model | In vitro purified protein complexes; crystallization of isolated coatomer subcomplexes. | Not Specified | Coatomer subunits, specifically COPI coat proteins: α‑COP, β′‑COP, and ɛ‑COP. | Protein purification, limited proteolysis, co‑expression of subcomplexes, crystallization, and X‑ray crystallography of protein complexes. | X‑ray crystallography for structural determination (multiwavelength anomalous diffraction and/or molecular replacement; structural model building and refinement) of the αβ′‑COP and αɛ‑COP subcomplexes. | Reveals conserved architectural principles among COPI, COPII, and clathrin vesicle coats, demonstrating a shared evolutionary origin and providing a structural framework for understanding vesicle formation and cargo sorting | 10.1016/j.cell.2010.05.030 |
|  | Rizzo et al. | 2021 | *In vitro* human cell systems — primarily HeLa cells; also primary human fibroblasts (PHFs) | Cell culture (mammalian cell lines). | Not Specified | GOLPH3 (Golgi Phosphoprotein 3), a Golgi‐localized protein acting in intra‐Golgi trafficking | Genetic manipulation: knockdown (KD) or overexpression (OE) of GOLPH3; expression of tagged proteins; siRNA interference; CRISPR/Cas9 knockouts in some contexts; biochemical pulldowns; toxin binding assays. | Immunofluorescence microscopy; cryo‐immunoelectron microscopy; flow cytometry (GSL detection with bacterial toxins); peptide pull‐down and co‐precipitation; lipidomics; synchronized trafficking assays using RUSH system; Western blotting; quantitative microscopy and line‐scan analyses. | GOLPH3 binds specific glycosphingolipid (GSL) biosynthetic enzymes through a cytosolic LxxR motif, sorting them into COPI vesicles for retrograde intra-Golgi transport. Acting within the Golgi cisternal maturation process, GOLPH3 controls enzyme localization and retention in the trans-Golgi and prevents their lysosomal degradation. Elevated GOLPH3 increases complex GSL synthesis, alters plasma membrane composition, and promotes mitogenic signaling via pathways such as Akt/mTOR, thereby supporting cell growth. | 10.15252/embj.2020107238 |
|  | Etienne et al. | 2005 | Human pancreatic duct CFPAC-1 cells (CFTR ΔF508 mutation) and CFPAC-1 cells transfected with wild-type CFTR to generate a “reverted” phenotype expressing normal CFTR. | In vitro | Not Specified | FTR (Cystic Fibrosis Transmembrane Conductance Regulator), specifically the effect of wild-type CFTR expression vs mutant ΔF508 CFTR on trafficking of other proteins through the secretory pathway. | Genetic transfection of wild-type CFTR into CFPAC-1 cellsWild-type CFTR was genetically transfected into CFPAC-1 cells to restore CFTR function, followed by a comparative analysis of cellular ultrastructure and Golgi compartment distribution. Differences between mutant CFTR cells and CFTR-restored cells were assessed to determine whether CFTR reconstitution rescues Golgi organization and associated trafficking defects. | Golgi organization and secretory pathway integrity were evaluated using immunocytochemistry for Golgi and secretory markers, electron microscopy to assess Golgi morphology and ultrastructure, and colocalization analyses of carbonic anhydrase IV trafficking as a functional marker of secretory pathway integrity. | CFPAC-1 cells expressing ΔF508 CFTR exhibit fragmented and dispersed Golgi stacks accompanied by impaired intracellular protein trafficking, including defective routing of carbonic anhydrase IV. Reintroduction of wild-type CFTR restores normal Golgi architecture, microtubule organization, and secretory pathway function, highlighting a critical role for CFTR in maintaining biosynthetic and secretory pathway integrity in pancreatic duct cells | doi.org/10.1369/jhc.4A6587.2005 |
|  | Dahan et al. | 2005 | Primary pancreatic acinar cells isolated from rodents (ex vivo cell model) stimulated with secretagogues such as cholecystokinin (CCK) to mimic physiologic secretory stimulus. | Cell culture of isolated acinar cells maintained in appropriate physiological medium; conditions mimicking secretagogue stimulation. | Not Specified | Dynamin (vesicle severing protein) and Golgi/TGN markers | Agonist stimulation with cholecystokinin (CCK) + dynamin manipulation | Immunofluorescence, live‑cell imaging, Golgi & TGN marker distribution, dynamin recruitment assays | CCK‑induced secretory stimulation causes selective fragmentation/vesiculation of the trans‑Golgi network (TGN) correlating with increased cytoplasmic Ca²⁺, and dynamin is recruited to the Golgi where it promotes TGN fragmentation, suggesting dynamic regulation of Golgi architecture during regulated secretion | 10.1053/j.gastro.2005.10.005 |
| Ubiquitin-Mediated Degradation Pathways | Younger et al. | 2006 | Cultured mammalian cells (HEK293 transfected with CFTR and mutants) | Cell culture | Not Specified | CFTR (wild‑type and ΔF508) and associated QC proteins (RMA1, Ubc6e, Derlin‑1, CHIP/Hsc70) | Overexpression and siRNA knockdown of QC components; proteasome inhibitor treatments | Western blotting, pulse‑chase labeling, co‑immunoprecipitation, fluorescence microscopy | Identified sequential ER quality‑control checkpoints involving ER membrane‑associated RMA1/Ubc6e/Derlin‑1 complex cooperating with cytosolic Hsc70/CHIP to recognize and target misfolded CFTR, especially ΔF508, for proteasomal degradation. RMA1 acts cotranslationally while CHIP acts post‑translationally. Derlin‑1 helps retain misfolded CFTR in ER. This defines how cells triage misfolded CFTR for degradation | 10.1016/j.cell.2006.06.041 |
|  | Tsukasa et al. | 2010 | Human cell lines (e.g., HeLa and IB3 cells) expressing rescued ΔF508-CFTR; post‑Golgi compartments studied in vitro and in cultured cells | Cell culture (mammalian cells in vitro) | Not Specified | Cystic Fibrosis Transmembrane Conductance Regulator (CFTR), specifically the ΔF508 mutant (rΔF508CFTR) | siRNA screening to deplete chaperones/co‑chaperones and ubiquitin ligases; in vitro ubiquitination assays; temperature‑dependent rescue of ΔF508CFTR | Functional siRNA screening; ELISA for plasma membrane (PM) stability and internalization; immunoblotting; in vitro ubiquitination assays; co‑immunoprecipitation | Shows that unfolded CFTR that escapes the ER is recognized and removed at the plasma membrane by peripheral quality-control mechanisms; highlights an additional layer beyond ERAD that limits functional CFTR at the cell surface | 10.1126/science.1191542 |
|  | Jarosch et al. | 2002 | *Saccharomyces cerevisiae* (yeast) cellular system, studying ER‑associated protein degradation pathways | Yeast cells, biochemical cell extracts, and in vivo degradation assays (proteasome/ubiquitin system context) | Not Specified | Retrotranslocation substrates of ERAD (e.g., model misfolded proteins such as CPY* in yeast), focusing on ubiquitinated ER proteins and their extraction factors | Genetic perturbation of ubiquitination machinery and functional analysis of AAA‑ATPase Cdc48; manipulation of ubiquitin chain formation on substrates; testing requirements for dislocation. | *In vivo* yeast genetics, biochemical ubiquitination assays, protein degradation and retrotranslocation detection (immunoblotting assays, monitoring ER‑associated degradation efficiency) | Demonstrates that ER protein dislocation requires polyubiquitination and the AAA-ATPase Cdc48, providing mechanistic insight into the retrotranslocation step of ERAD | 10.1038/ncb746 |
|  | Linhui et al. | 2022 | Human pancreatic cancer cell lines (e.g., Panc-1, PaTu-8988), 293T cells, and xenograft mouse models | In vitro cell culture (DMEM with 10% FBS), and in vivo nude mice xenograft tumors | 5 mice per group in xenograft assays for tumor growth experiments (as described in methods) | Fbxo45 (F-box protein, E3 ubiquitin ligase component) and USP49 (ubiquitin-specific protease) | Genetic overexpression and knockdown (siRNA) of Fbxo45 and USP49; proteasome inhibition (MG132); cycloheximide chase; mutation and truncation constructs; xenograft mouse tumor models | Co‑immunoprecipitation, Western blot, ubiquitination assays, qRT‑PCR, wound‑healing & transwell assays, MTT proliferation assay, TUNEL apoptosis assay, immunohistochemistry | Fbxo45 interacts with and ubiquitinates USP49 via its SPRY domain, promoting degradation of USP49, enhancing pancreatic cancer cell proliferation, migration, invasion and tumor growth in vivo; Fbxo45 expression is negatively correlated with USP49 and associated with poorer survival | 10.1038/s41419-022-04675-2 |
| Endocytic Recycling and Membrane | Heda GD et al. | 2001 | Polarized epithelial cell culture (MDCK cells expressing wild-type or ΔF508-CFTR) | In vitro cell culture | Not Specified | CFTR (Cystic Fibrosis Transmembrane Conductance Regulator), specifically ΔF508-CFTR | Stable expression of wild-type or ΔF508-CFTR; metabolic pulse–chase labeling to assess CFTR turnover at the plasma membrane | Pulse–chase analysis with radiolabeling, cell surface biotinylation, immunoprecipitation, and SDS–PAGE/autoradiography | ΔF508-CFTR that reaches the plasma membrane in polarized epithelial cells exhibits a significantly shortened biochemical half-life compared with wild-type CFTR, indicating enhanced post-Golgi instability and accelerated removal from the cell surface. This defect contributes to reduced functional CFTR at the apical membrane in cystic fibrosis | 10.1152/ajpcell.2001.280.1.C166 |
|  | Sharma et al. | 2004 | Mammalian cell culture (primarily human epithelial cell lines expressing CFTR, including wild-type and ΔF508-CFTR) | In vitro cell culture system | Not Specified | CFTR (Cystic Fibrosis Transmembrane Conductance Regulator); involvement of endosomal sorting and quality-control machinery (e.g., ubiquitination-dependent sorting, ESCRT-related pathways) | Genetic expression of wild-type vs. misfolded (ΔF508) CFTR, temperature rescue, pharmacological inhibition of lysosomal and proteasomal degradation pathways, and trafficking perturbations | Surface biotinylation, pulse-chase analysis, immunoprecipitation, confocal microscopy | Misfolded CFTR that escapes the ER quality-control system is rapidly removed from the plasma membrane and diverted from the recycling pathway to lysosomal degradation. In contrast, properly folded CFTR efficiently recycles back to the cell surface. This demonstrates the existence of a post-ER peripheral quality-control mechanism that recognizes misfolded CFTR and targets it for degradation, contributing significantly to CFTR loss in cystic fibrosis. | 10.1083/jcb.200312018 |
|  | Bomberger et al. | 2009 | Human airway epithelial cells (CFBE cells) | Polarized airway epithelial cell culture | Not Specified | Cystic Fibrosis Transmembrane Conductance Regulator (CFTR) | Activity-based screen for deubiquitinating enzymes; siRNA-mediated knockdown of USP10; overexpression of wild-type and dominant-negative USP10 | Chemical proteomic screening, RNA interference (siRNA), overexpression constructs, Western blotting, immunoprecipitation, ubiquitination analysis, chloride secretion measurement | USP10 localizes to early endosomes and deubiquitinates CFTR, promoting its recycling back to the plasma membrane. Knockdown of USP10 increases CFTR ubiquitination and lysosomal degradation and decreases CFTR at the apical membrane and CFTR-mediated chloride secretion, while overexpression of wild-type USP10 enhances CFTR abundance and reduces its ubiquitination. | 10.1074/jbc.M109.001685 |
| Regulatory Interactors Modulating CFTR Function | Angyal et al. | 2024 | Adult stem cell‑derived organoids from porcine pancreas, differentiated into ductal epithelial monolayers | Porcine pancreatic ductal organoid cultures and epithelial monolayers in vitro | Not Specified | CFTR (cystic fibrosis transmembrane conductance regulator) | Exposure of ductal epithelial monolayers to pro‑inflammatory cytokines (IL‑1β, IL‑6, IFN‑γ, and TNF‑α), alone and in combination | Electrophysiological measurement of anion secretion (Ussing chamber short‑circuit current), immunocytochemistry, expression analysis | Shows that pro-inflammatory cytokines enhance CFTR-dependent anion (Cl⁻ and HCO₃⁻) secretion, indicating that inflammation can modulate CFTR function in pancreatic ducts and potentially influence pancreatic pathology | doi.org/10.1186/s11658-024-00537-1 |
|  | Constanze et al. | 2007 | Isolated rat pancreatic acinar cells | Cultured acinar cells stimulated in vitro with secretagogues | Not specified in abstract (detailed cell numbers/biological replicates reported in full text) | Markers of ER stress/UPR: BiP (chaperone), PERK phosphorylation, XBP1 splicing, CHOP expression | Stimulation with secretagogues: CCK8 (10 pM–10 nM), JMV‑180 (10 nM–10 μM), bombesin (1–100 nM) | Measurement of UPR components (BiP, PERK, XBP1, CHOP) and amylase secretion & intracellular trypsin activity assays (biochemical and molecular analyses) | All secretagogues increased BiP and XBP1 splicing, activating adaptive ER stress, whereas only high-dose CCK8 triggered PERK | 10.1152/ajpgi.00078.2007 |
