# Supplementary material for: Protein sorting and proteostasis mechanisms in CFTR-related exocrine pancreas dysfunction: A systematic narrative review
Source: Channels (Austin). 2026 Sep 12;20(1):2731660. doi: 10.1080/19336950.2026.2731660 (PMC13577266; doi:10.1080/19336950.2026.2731660)
Supplement: Supplementary file 4 [file KCHL_A_2731660_SM0086.docx]

**Detailed Search Strategy**

A comprehensive literature search was conducted in PubMed/MEDLINE, Scopus, and Wiley Online Library to identify studies investigating CFTR channelopathies associated with abnormalities in protein sorting, processing, trafficking, and quality-control machinery within the exocrine pancreas. The search covered studies published from January 1, 2000 to November 30, 2025 and was limited to English-language publications. Controlled vocabulary and free-text terms were combined using Boolean operators AND and OR, with database-specific subject headings, phrase searching, and field codes applied where applicable.

The search strategy incorporated four main concepts:

1. CFTR and CFTR dysfunction/channelopathies;
2. Protein sorting, processing, folding, trafficking, and degradation machinery;
3. The exocrine pancreas and pancreatic secretory function; and
4. Experimental study models.

Core CFTR-related terms included: “CFTR,” “CFTR mutations,” “CFTR channelopathies,” “cystic fibrosis transmembrane conductance regulator,” “CFTR misfolding,” and “CFTR trafficking defects.” To capture the role of intracellular sorting systems, we used protein-processing terms such as “protein sorting,” “protein trafficking,” “endoplasmic reticulum,” “Golgi apparatus,” “ER-associated degradation (ERAD),” “chaperone proteins,” “COPII vesicles,” “quality control machinery,” and “ubiquitin-proteasome pathway.” Because the focus is on the exocrine pancreas, organ-specific keywords were included: “exocrine pancreas,” “pancreatic acinar cells,” “pancreatic ductal cells,” “pancreatic secretion,” and “bicarbonate secretion.” To ensure broad coverage of experimental approaches, additional search terms were incorporated for study models such as “animal models,” “mouse models,” “rat models,” “organoids,” “cell lines,” and “*in vitro* studies.”

| **Database** | **Search string** | **Date searched** |
| --- | --- | --- |
| PubMed/MEDLINE | ("CFTR"[Mesh] OR "CFTR"[tiab] OR "cystic fibrosis transmembrane conductance regulator"[tiab] OR "CFTR mutations"[tiab] OR "CFTR channelopathies"[tiab] OR "CFTR misfolding"[tiab] OR "CFTR trafficking defects"[tiab]) AND ("Protein Transport"[Mesh] OR "protein sorting"[tiab] OR "protein trafficking"[tiab] OR "protein processing"[tiab] OR "protein folding"[tiab] OR "endoplasmic reticulum"[tiab] OR "Golgi apparatus"[tiab] OR "ER-associated degradation"[tiab] OR ERAD[tiab] OR "chaperone proteins"[tiab] OR chaperone[tiab] OR "COPII vesicles"[tiab] OR COPII[tiab] OR "quality control machinery"[tiab] OR "ubiquitin-proteasome pathway"[tiab] OR ubiquitin[tiab]) AND ("exocrine pancreas"[tiab] OR "pancreatic acinar cells"[tiab] OR "pancreatic ductal cells"[tiab] OR "pancreatic secretion"[tiab] OR "bicarbonate secretion"[tiab] OR pancreas[tiab]) AND ("animal models"[tiab] OR "mouse models"[tiab] OR "rat models"[tiab] OR organoids[tiab] OR "cell lines"[tiab] OR "in vitro"[tiab]) | December 2025 |
| Scopus | TITLE-ABS-KEY ("CFTR" OR "cystic fibrosis transmembrane conductance regulator" OR "CFTR mutation*" OR "CFTR channelopath*" OR "CFTR misfold*" OR "CFTR trafficking defect*") AND TITLE-ABS-KEY ("protein sorting" OR "protein trafficking" OR "protein processing" OR "protein folding" OR "protein maturation" OR "protein quality control" OR "endoplasmic reticulum" OR "ER-associated degradation" OR ERAD OR "Golgi apparatus" OR "vesicular trafficking" OR "COPII" OR "chaperone*" OR ubiquitin* OR proteasome* OR autophagy OR lysosome* OR "endosomal trafficking" OR "membrane trafficking" OR "apical targeting") AND TITLE-ABS-KEY ("exocrine pancreas" OR pancreas OR pancreatic OR "pancreatic acinar cell*" OR "pancreatic ductal cell*" OR "pancreatic duct*" OR "pancreatic secretion" OR "bicarbonate secretion") AND TITLE-ABS-KEY ("animal model*" OR "mouse model*" OR "murine model*" OR "rat model*" OR "rodent model*" OR organoid* OR "cell line*" OR "primary cell*" OR "cell culture" OR "in vitro" OR "in vivo") | December 2025 |
| Wiley Online Library | TITLE-ABS-KEY ("CFTR" OR "cystic fibrosis transmembrane conductance regulator" OR "CFTR mutation*" OR "CFTR channelopath*" OR "CFTR misfold*" OR "CFTR trafficking defect*" OR "CFTR processing defect*") AND TITLE-ABS-KEY ("protein sorting" OR "protein trafficking" OR "protein processing" OR "protein folding" OR "protein maturation" OR "protein quality control" OR "endoplasmic reticulum" OR "ER-associated degradation" OR ERAD OR "chaperone protein*" OR chaperone* OR "COPII vesicle*" OR COPII OR "Golgi apparatus" OR "Golgi trafficking" OR "vesicular trafficking" OR "membrane trafficking" OR "endosomal trafficking" OR "apical targeting" OR "ubiquitin-proteasome pathway" OR ubiquitin* OR proteasome* OR autophagy OR lysosome*) AND TITLE-ABS-KEY ("exocrine pancreas" OR pancreas OR pancreatic OR "pancreatic acinar cell*" OR "pancreatic ductal cell*" OR "pancreatic duct*" OR "pancreatic secretion" OR "bicarbonate secretion") | December 2025 |
